# Hydroxycinnamic Acid Extraction from Multiple Lignocellulosic Sources: Correlations with Substrate Composition and Taxonomy for Flavoring and Antioxidant Applications

**DOI:** 10.1021/acs.jafc.4c08540

**Published:** 2024-12-05

**Authors:** Robson Tramontina, Eupidio Scopel, Victor Gustavo Kelis Cardoso, Manoela Martins, Marcos Fellipe da Silva, Bárbara Flaibam, Marcos J. Salvador, Rosana Goldbeck, André Damasio, Fabio Marcio Squina

**Affiliations:** ^†^Departamento de Biologia Vegetal (DBV) ^‡^Departamento de Biologia Funcional e Molecular (BFM), Universidade Estadual de Campinas (UNICAMP), Campinas, São Paulo 13083-859, Brazil; b Laboratório de Ciências Moleculares, Universidade de Sorocaba (UNISO), Sorocaba, São Paulo 18023-000, Brazil; c Instituto de Química, Universidade Estadual de Campinas (UNICAMP), Campinas, São Paulo 13083-970, Brazil; d Escola de Engenharia de Alimentos, Universidade Estadual de Campinas (UNICAMP), Campinas, São Paulo 13083-862, Brazil

**Keywords:** industrial crops, hydroxycinnamic acid, chemometrics, lignin valorization, biotransformation

## Abstract

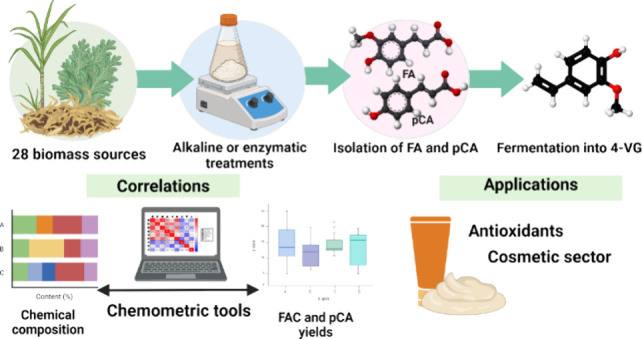

The extraction of hydroxycinnamic acids (HCADs) is a strategy for lignocellulosic biomass valorization due to their high value-added nature and the possibility of application as flavoring and antioxidants. This study proposes correlations between the composition and taxonomy of 28 globally available agro-industrial feedstocks with the production of HCADs using chemometric tools. Principal component analysis indicated strong correlations between ferulic acid release and hemicellulose type and content, especially in grass biomasses. Conversely, *p-*coumaric acid release was mainly correlated with cellulose content across diverse taxonomic origins. Among the evaluated agro-industrial feedstocks, corn-based biomasses were identified as prime sources of ferulic acid after mild alkaline treatment, releasing up to 10.5 g kg^–1^ and producing hydrolysates with an antioxidant capacity up to 3.3 mmol Trolox equivalents g^–1^. Notably, sugar cane bagasse was the best source of *p*-coumaric acid, yielding 4.8 g kg^–1^. Corn hydrolysates were successfully converted into 4-vinylguaiacol using a genetically modified *Saccharomyces cerevisiae* strain, achieving high yields of 0.75 g L^–1^. This work enhances our understanding of HCAD sources and biomass valorization strategies, demonstrating potential applications in the food and cosmetics sectors.

## Introduction

Lignocellulosic biomasses are renewable sources for producing energy and bioproducts, including chemicals and materials, due to their abundant availability and cost-effectiveness.^1^ The lignocellulosic biomass potential encompasses the variety of available feedstocks, including those obtained as residues from the agro-industrial sector, such as residues from corn, sugar cane, and rice processing.^2^

Despite their abundant availability and biotechnological potential, the utilization of lignocellulosic substrates is still scarce for producing high-value-added products. Most of these biomass materials are primarily used in less value-added applications, such as for heat generation or biofuel production, which limits the potential for broadening the bioproduct portfolio.^3,4^ Additionally, several agro-industrial biomasses are less investigated for producing renewable chemicals, such as elephant grass, coffee husk, eucalyptus bark, miscanthus, and peanut hull, despite being rich biotechnological substrates that can be potentially used in biorefineries. A significant barrier to the utilization of lignocellulosic substrates is their intricate composition and morphology.^5^ These drawbacks require careful evaluations to develop specific processes and potential applications for each available biomass.^6^

Hydroxycinnamic acids (HCADs), specifically ferulic and coumaric acids (FA and pCA, respectively), are components of lignocellulosic biomass. HCADs are responsible for augmenting the mechanical integrity of plants by cross-linking plant cell wall polymers.^7^ HCADs have potential direct applications, ranging from antimicrobials and antioxidants to UV radiation absorption, representing a global market demand projected to reach USD 602 million by 2029.^8^ In addition, HCADs are interesting platform molecules and can be converted into high-value-added chemicals through biochemical or chemical pathways. One of the possible molecules include 4-vinyl guaiacol (4-VG), which is a flavoring component. The possibilities of HCAD use highlight their importance in the chemical industry and can potentially add value to biomass feedstocks.^9,10^

Historical research tracing back decades has explored the roles of phenolic acids in plant cell walls.^11^ However, challenges persist, and the variability of HCAD distribution across different plant species and anatomical locations remains remarkable.^12,13^ For instance, distinct human-cultivated crops, such as rice, wheat, and corn, are commelinid monocots of the *Poales* order and exhibit substantial HCAD content in their compositions.^6^ While numerous reports provide data on the percentage of HCADs in agro-industrial residues, the literature needs comprehensive studies comparing these feedstocks under uniform experimental conditions and parameters within a single experimental setup.^14^ This gap hinders a direct comparison and understanding of HCAD distribution across agro-industrial residues.

Alkaline and enzymatic methods are commonly used for the HCAD extraction. Enzymes such as glycoside hydrolases and feruloyl esterases offer clean and selective extraction of the components in milder conditions. On the other hand, alkali solutions are favored for their speed, simplicity, and cost-effectiveness.^14^ Therefore, the value proposition of agro-industrial byproducts is accentuated by converting them into an array of products using sustainable processes, which depends on the extraction methodology.

We conducted an in-depth study to evaluate and compare 28 biomass substrates for HCAD production. Our study aims to understand the main correlations between biomass composition and taxonomy with HCAD content to establish sustainable biomass sources for HCAD production and application, such as in the cosmetic sector and flavor production. Principal component analysis was applied to analyze the main components of potential feedstocks, specifically cellulose, lignin, and hemicellulose, and their relationships with HCAD extraction using enzymatic or alkaline hydrolysis methods. After identifying the most promising HCAD sources, we evaluated the inherent antioxidant properties of their HCAD extracts and HCAD bioconversion into the valuable flavor compound 4-VG.

## Experimental Section

### Feedstock Preparation and Chemicals

Twenty-eight plant biomass feedstocks were utilized in this study. Details of which, encompassing their lignocellulosic composition, origin, and previous citations in which their chemical composition was described, are presented in Table 1 as reference. Most feedstocks were categorized as crop byproducts, but we also considered nonfeed agro-industrial residues with partially purified biomass components. Raw biomasses were subjected to a standardized size-reduction protocol to approximately 0.5 cm in length and 1.0 mm in thickness in a disintegrator (DM 540, IRBI, São Paulo, Brazil). After this processing, the conditioned biomass samples exhibited an average moisture content below 10% (w/w). All chemicals used as analytical standards were purchased from Sigma-Aldrich.

**Table 1:** Comparative Analysis of FA and pCA Hydrolysate Concentrations in mmol^–1^ and Biomass Composition (% w/w) across Various Biomass Types Subjected to Mild-Alkaline and XynZ Enzymatic Treatments

		**alkaline treatment**		**enzymatic treatment**		**biomass composition**					
	**biomass**	**FA** (mmol L^–1^)	**pCA** (mmol L^–1^)	**FA** (mmol L^–1^)	**pCA** (mmol L^–1^)	**cellulose (%)**	**hemicellulose (%)**	**lignin (%)**	**extractives (%)**	**ash/others (%)**	**reference**
1	*Agave sisalana* leaves	n.d.	0.10 ± 0.01	n.d.	n.d.	20.3 ± 0.8	8.7 ± 0.6	9 ± 2	51.9 ± 0.6	11.3 ± 0.6	(30)
2	annatto bran	0.01 ± 0.01	0.01 ± 0.01	0.02 ± 0.01	0.02 ± 0.01	28.3 ± 0.2	6.0 ± 0.1	24.9 ± 0.3	19.9 ± 0.1	5.6 ± 0.2	this work
3	barley	0.71 ± 0.03	0.60 ± 0.02	0.04 ± 0.01	0.01 ± 0.01	17.9 ± 0.1	35.7 ± 0.1	17.9 ± 0.4	9.03 ± 0.4	3.7 ± 0.1	this work
4	cocoa husks	0.03 ± 0.01	n.d.	0.04 ± 0.01	0.06 ± 0.01	22.5 ± 0.4	11.9 ± 0.2	30.1 ± 0.9	9.8 ± 0.9	7.6 ± 0.2	this work
5	coffee husk	0.04 ± 0.01	0.03 ± 0.01	0.02 ± 0.01	0.02 ± 0.01	27.8 ± 0.8	24.7 ± 0.6	36 ± 2	10.8 ± 0.8	1.0 ± 0.6	(39)
6	corn cob	3.2 ± 0.1	4.9 ± 0.4	0.04 ± 0.01	0.04 ± 0.01	30.9 ± 0.3	26.7 ± 0.7	11.6 ± 0.4	23.3 ± 0.8	4.5 ± 0.2	this work
7	corn distiller’s grains	2.5 ± 0.1	0.18 ± 0.01	0.26 ± 0.02	0.06 ± 0.01	5.3 ± 0.1	31.1 ± 0.3	6 ± 1	18 ± 1	3.9 ± 0.1	this work
8	corn gluten feed	4.0 ± 0.2	0.07 ± 0.01	0.32 ± 0.02	0.38 ± 0.04	22.3 ± 0.2	27 ± 1	12.4 ± 0.2	15 ± 2	1.2 ± 0.1	this work
9	corn husk	1.8 ± 0.1	1.3 ± 0.1	0.22 ± 0.02	0.22 ± 0.02	35.2 ± 0.4	20 ± 2	22.2 ± 0.1	13 ± 1	5.8 ± 0.1	this work
10	corn pericarp	4.2 ± 0.1	0.20 ± 0.02	0.28 ± 0.01	0.08 ± 0.01	15 ± 3	40 ± 2	3 ± 1	38 ± 3	0.9 ± 0.1	(10)
11	cottonseed	0.06 ± 0.01	0.07 ± 0.01	0.04 ± 0.01	0.04 ± 0.01	12.2 ± 0.1	14 ± 1	41 ± 2	25 ± 0.9	3.8 ± 0.1	this work
12	elephant grass	1.7 ± 0.2	0.73 ± 0.02	0.12 ± 0.01	0.04 ± 0.01	30 ± 2	24 ± 2	22.5 ± 0.1	20.2 ± 0.2	5.7 ± 0.1	(40)
13	eucalyptus bark	0.10 ± 0.01	0.27 ± 0.01	0.02 ± 0.01	0.06 ± 0.01	43 ± 2	16.2 ± 0.5	35.1 ± 0.1	3.2 ± 0.3	0.4 ± 0.1	this work
14	grape peel	n.d	0.85 ± 0.06	0.02 ± 0.01	0.04 ± 0.01	23.0 ± 0.9	4.5 ± 0.9	29 ± 2	15 ± 3	12.5 ± 0.7	this work
15	grape seed	0.08 ± 0.01	n.d.	0.04 ± 0.01	n.d.	10.8 ± 0.5	8.6 ± 0.6	42.2 ± 0.4	24 ± 2	4.8 ± 0.8	this work
16	green value lignin	2.2 ± 0.1	0.28 ± 0.03	n.d.	n.d.			100.0			(41)
17	*Miscanthus sacchariflorus* leaves	1.4 ± 0.1	2.0 ± 0.2	0.04 ± 0.01	0.44 ± 0.01	39.1 ± 0.9	15.1 ± 0.7	16 ± 1	23.8 ± 0.8	4.0 ± 0.3	this work
18	peanut hull	n.d.	0.17 ± 0.01	n.d.	0.02 ± 0.01	11.2 ± 0.4	18 ± 1	20 ± 2	1.5 ± 0.2	6.6 ± 0.1	this work
19	*Plantago psyllium*	0.01 ± 0.01	0.01 ± 0.01	0.02 ± 0.01	n.d.	18.0 ± 0.4	13.8 ± 0.1	46 ± 3	18.2 ± 0.8	1.0 ± 0.1	this work
20	rice bran	0.84 ± 0.03	0.42 ± 0.01	0.12 ± 0.01	0.02 ± 0.01	28.6 ± 0.4	13.9 ± 0.1	41 ± 1	5.9 ± 0.6	7 ± 1	this work
21	rice husk	0.02 ± 0.01	0.96 ± 0.05	0.04 ± 0.01	0.14 ± 0.01	28.6 ± 0.1	16.6 ± 0.1	33.1 ± 0.3	8.5 ± 0.2	14.8 ± 0.2	(42)
22	rice straw	0.59 ± 0.01	0.47 ± 0.02	n.d.	0.14 ± 0.01	38.7 ± 0.1	18.9 ± 0.1	25.5 ± 0.3		16.2 ± 0.1	this work
23	soybean meal	n.d.	0.01 ± 0.01	n.d.	n.d.	6.9 ± 0.3	20.4 ± 0.6	10.0 ± 0.2	49.3 ± 0.1	6.3 ± 0.1	this work
24	sugar cane bagasse	0.67 ± 0.02	2.9 ± 0.1	0.18 ± 0.01	0.62 ± 0.02	40.1 ± 0.9	27.5 ± 0.7	18.5 ± 0.8	5.4 ± 0.1	5.8 ± 0.1	(44)
25	sugar cane straw	0.52 ± 0.04	1.3 ± 0.1	0.12 ± 0.01	0.14 ± 0.02	39 ± 1	27.6 ± 0.4	21.4 ± 0.9	2 ± 1	1.3 ± 0.2	(45)
26	wheat straw	0.81 ± 0.06	0.49 ± 0.03	n.d.	0.10 ± 0.01	38.0 ± 0.4	24.0 ± 0.1	19 ± 3	11.0 ± 0.8	1.0 ± 0.1	(9)
27	wheat straw organosolv lignin	0.28 ± 0.01	0.27 ± 0.02	n.d.	n.d.			100.0			(46)
28	wheat arabinoxylan	0.71 ± 0.06	0.01 ± 0.01	0.12 ± 0.01	0.02 ± 0.01		95.0	0.1	0.0	2.0	(47)

### Biomass Processing by Alkaline Extraction of HCADs

The feedstocks shown in Table 1 were treated under uniform conditions with a mild alkaline process using a solution of 0.5 mol L^–1^ NaOH in a solid: liquid ratio of 10 wt % at 85 °C in a water bath without agitation for 2 h. After the treatment, approximately 10 mL of alkaline liquors were generated from 1 g of each biomass.

### Biomass Processing by Feruloyl Esterase (XynZ) Extraction of HCADs

Enzymatic treatments were carried out using the recombinant enzyme XynZ, composed of a GH10 xylanolytic and a C-terminal domain with feruloyl esterase activity produced according to Tramontina et al. (2023).^15^ Before the XynZ treatment, 0.5 g of each feedstock was buffered with sodium phosphate buffer 0.1 mol L^–1^ at pH 5.4 with a final volume of 10 mL (5 wt %). The XynZ treatment was conducted in a 50 mL hydrolysis flask using 0.5 mg of purified enzyme at 50 °C for 48 h at 10 rpm in a hybridization shaker (RPN2511 Amersham, São Paulo, Brazil). Additional experiments were carried out to understand the enzymatic release of HCADs from corn-related biomasses. For this, the substrates were also hydrolyzed with XynZ supplemented with lignocellulosic cocktails (Cellic CTec 2 and Cellic HTec - *Novonesis*) with a protein loading of 25 mg of each and XynZ under the same conditions described above. The HCAD release degree of synergism (DS) [(XynZ + Cocktail)/XynZ] was calculated.^16^

### HCAD Isolation

The alkaline liquor produced after each feedstock treatment and the supernatants after enzymatic treatment had their pH adjusted to 2 with concentrated HCl (37 wt %) and centrifuged at 3000*g* for 15 min. The liquid fraction was kept at 4 °C until further use. In experiments in which purifications of HCADs in the hydrolysates were necessary, we purified them by using liquid–liquid extraction with 2:1 ethyl acetate. The organic fraction separated from the aqueous phase was dried in a rotary evaporator (Z319651 – Merck, São Paulo, Brazil) at 80 °C. The dried powders obtained were weighed and further stored for analysis.

### FA Biotransformation to 4-VG via Yeast Fermentation

The industrial *Saccharomyces cerevisiae* Ethanol Red strain expressing the cofactor-free phenolic acid decarboxylase (PDC) enzyme (PDC_B5a) from the ligninolytic strain *Rhodosporidium fluviale* LM-2 was used in this work to convert FA, extracted from the lignocellulose hydrolysates, into 4-VG.^17^*S. cerevisiae* Ethanol Red, without any modification, was employed in the same experiment as a control. A colony from the YPD plate (1% yeast extract, 2% peptone, and 2% glucose) was picked up and precultivated in a 250 mL Erlenmeyer flask with 80 mL of YPD medium at 30 °C for 16 h in an orbital shaker (Multitron – INFORS, São Paulo, Brazil). Then, the preculture was centrifuged at 4000 rpm for 10 min. The cell pellet was used for inoculation in 40 mL of the YP medium (approximately 5g L^–1^ cell dry weight (CDW) containing 80% (v/v) plant biomass hydrolysates. The cultivations were incubated at 30 °C and 250 rpm for 48 h. The kinetic parameter calculations were performed according to Santos et al. (2016).^18^ Aliquots were collected during the experiment, and the supernatant samples were kept at 4 °C until further analyses were performed.

### Chemical Composition of Lignocellulosic Biomasses

Cellulose, hemicellulose, lignin, extractives, and ashes contents were determined according to the National Renewable Energy Laboratory (NREL) methodologies.^19^ Some biomasses had their chemical composition previously determined according to Table 1. Overall, sugars, organic acids, and degradation products were converted to their respective percentage of polysaccharides. The ash content was determined by burning them to determine the content of inorganic materials using a furnace, and extractives were quantified by Soxhlet extractions with cyclohexane + ethanol solution (1:1), except when indicated in the respective references indicated in Table 1.

### Phenolic Quantification

Phenolic content was determined by reverse-phase high-performance liquid chromatography (RC-HPLC – Dionex UltiMate3000 – Thermo Scientific, United States) using a diode arrange detector. The absorbance of the samples was measured at 280 nm, according to Martins et al. (2022).^10^ Before all HPLC analyses, the samples were filtered in 0.45 μm nylon syringe filters. A calibration curve (0.01–5.00 mmol L^–1^) of the phenolic standards was used to calculate the sample concentrations. The chromatographic peaks were analyzed using the Chromeleon7 software version 7.2.5.9507 (Thermo Fisher Scientific, United States).

### Nitrogen Content Determined by Elemental Analysis for Protein Quantification

The nitrogen content of the plant biomass samples was determined using a CHN elemental analyzer (PerkinElmer CHN2400, United States). The protein content of each sample was estimated by converting the nitrogen content using the factor of 6.3, based on the assumption that proteins contain 16% nitrogen on average.^20^

### Antioxidant Capacity by the ORAC-FL Assay

The antioxidant capacity of the selected alkaline hydrolysate extracts was determined using the oxygen radical absorbance capacity (ORAC-FL) assay, employing fluorescein as the fluorescent probe and 2,2′-azobis(2-amidinopropane) dihydrochloride (AAPH) as the free radical source according to Salvador et al. (2006).^21^ Various dilutions of the samples (5.0 to 500.0 μg mL^–1^) were prepared in a phosphate buffer/DMSO solution (99:1, v/v). Trolox (6-hydroxy-2,5,7,8-tetramethylchroman-2-carboxylic acid), a known antioxidant, was used as a standard at 12.5–200.0 μmol L^–1^ concentrations. The measurements were performed using an Accuris SmartReader 96 microplate absorbance reader equipped with a fluorescent filter (excitation λ = 485 nm and emission λ = 528 nm) (Merck, Germany). The reaction was monitored every 2 min at 37 °C for a total duration of 70 min. The results were expressed as micromoles of Trolox equivalent (TE) per gram of dried extract (μmol TE g^–1^). FA, pCA, caffeic acid, chlorogenic acid, quercetin, and isoquercetin were utilized as positive controls, while the solvent was the negative control. All experiments were conducted in triplicate.

### Multivariate Analysis

Principal component analysis and Pearson correlation analysis were employed to identify similarities and correlations between the samples and their compositional information. Values of FA and pCA extracted after alkaline and enzymatic treatments, in addition to the biomass compositions (Table 1), were imported to MATLAB R2021b (MathWorks, United States) containing PLS_Toolbox 9.0 (Eigenvector, United States). Substrates that underwent previous fractionation of purification, including lignin and arabinoxylan, were removed from the data set since the main goal was to understand correlations with raw substrates. Also, the biomass component named “Extractives” was not evaluated under these analyses since its chemical composition is highly heterogeneous from one sample to another and can include diverse groups of molecules. The final data matrix (Table 1) depicts the information on the lignocellulosic materials (25 samples) and the FA, pCA, biomass, and other components. For Pearson correlation analysis, the coefficients were calculated to estimate linear relationships between variables based on the final matrix. The final matrix was autoscaled for the Principal Component Analysis model to remove the effects of magnitude differences before model calculations.

### Phylogenetic Analysis

A neighbor-joining phylogenetic tree with the maximum composite likelihood method and 1000 replicates of Bootstrap testing was constructed to elucidate the evolutionary relationships among the industrial crops studied.^22^ The data set comprised 24 nucleotide sequences, with 1345 positions analyzed postdeletion of ambiguous positions (Supplementary File 1: session 4). The analyses were performed in MEGA11 software (United States).

### Heatmap Visualization

Concentrations of FA and pCA were visualized in a heatmap overlaid on the phylogenetic tree using the iTOL database, with the average values of each crop found in Table 1. The ITS entries sequences with gray icons in Figure 2 (*Ginkgo biloba*, *Helianthus annuus*, *Solanum tuberosum*, *Pinus*, *Nymphaea*, *Musa*, and *Lilium*) were not evaluated toward HCAD content and were inserted in the analysis for a better evolutionary alignment across the plant kingdom.

### Statistical Analyses

All analyses in this work were performed in triplicate. Results were considered significant when the P value was less than 0.05.

## Results and Discussion

### Biomass Chemical Composition Displays the Diversity of Content, Function, and Types of Polymers in Lignocellulosic Biomasses

The first part of this work aims to screen biomass substrates for HCAD extraction and guide future biomass selections according to their botanical origin. It is considered that understanding the variability among these constituents is pivotal for optimal HCAD production. We selected 28 distinct feedstocks for this and evaluated HCAD extraction through chemical (alkaline) and enzymatic strategies.

In our data set, the cellulose content ranged from 6.9 wt % in soybean meal to 43.1 wt % in eucalyptus bark (Table 1), indicating the composition variability between the substrates. In terms of hemicelluloses, the content varied from 4.5 wt % in grape peels (composed of glucuronoarabinoxylan (GAX) + pectin) to 40.3% (w/w) in corn pericarp (CP) (mainly arabinoxylan [AX]).^10,23^

Unlike cellulose, which has a precise chemical structure, hemicelluloses can comprise various sugar moieties. In monocots, hemicelluloses like GAX and AX have a xylose backbone with α-L-arabinofuranosyl side chains cross-linked with lignin via FA.^13^ Accordingly, the cell wall structure in AX-rich biomasses is less dense in biomasses with lower lignin content, making these ester bonds more accessible for hydrolysis, especially under alkaline conditions.^24^ On the other hand, dicots exhibit a wider variety of hemicelluloses, such as xyloglucan, which has fewer HCADs.^25^

It is noteworthy that the CP, the kernel’s outermost layer, surpasses the corn husk (CH) content of hemicellulose, FA, and pCA release (Table 1), which has a likely anatomical reason. CP is rich in AX and FA, thus adapted to act as a robust shield, defending the kernel against potential threats like pathogens, desiccation, and oxidative stress.^26,27^ Therefore, the FA and pCA content variation among these biomasses may play distinctive roles in the plant structural framework: whereas FA is primarily linked to GAX through ester linkages, some pCA also connect to AX in young tissues.^24^

Regarding the lignin content of the lignocellulosic materials, the content spanned from 2.8% in CP to 42.2% in grape seed. CP possesses the lowest lignin content since the main composition of this biomass is AX, which is fundamental for its biological function in the maize grain.^26^ The specific composition of lignin in terms of its basic units is influenced by biomass botanical origin, and varies noticeably among hardwoods, softwoods, and grasses.^28^ In hardwoods, FA and pCA are primarily attached to the G-position of its structure, but in grasses, lignin is mainly modified with pCA.^12,24,28^ Likewise, the augmented lignin concentration in specific plant components, such as barks and seeds, likely mirrors their essential physiological roles in providing mechanical strength and defense against environmental challenges.^29^

Finally, extractives encompass a variety of biomass components with diverse compositions, including nonpolar substances such as fatty acids and sterols and polar substances such as starch and pectin. The data revealed a notable variation in extractive content, from a near absence in rice straw to 51.9% in *Agave sisalana* leaves due to abundant reserves of pectic compounds, β2–2/6-fructans, and callose (β-1,3-glucan).^30^

### HCAD Release from the Lignocellulosic Substrates

FA and pCA concentrations after alkaline and enzymatic treatments are shown in Table 1. The analysis showed that grasses emerged with the highest concentrations of HCADs (up to 1.0 mmol L ^–1^) among the plant biomasses evaluated in this study.

CP showed alkali-released HCAD concentrations of 4.4 ± 0.1 mmol L^–1^ and XynZ-released concentrations of 0.32 ± 0.03 mmol L^–1^, similar to CGF (4.0 ± 0.2 and 0.7 ± 0.2 mmol L^–1^ of alkaline and enzymatic treatments, respectively). Accordingly, concentrations of 0.66 mmol L^–1^ of FA had been reported for CGF hydrolysates.^31^ The CC exhibited 8.1 ± 0.4 and 0.04 ± 0.01 mmol L^–1^ for alkaline and enzymatic treatments, respectively, whereas previous reports found similar results.^32^ Furthermore, 2.7 ± 0.1 mmol L^–1^ for alkaline and 0.32 ± 0.02 mmol L^–1^ for enzymatic treatments revealed the potential of corn distiller grains (DDGS) as a source of HCADs that was not previously reported.

Considering the lignocellulose composition, both CGF and CP are characterized by their high AX content, constituting 40% and 27% (w/w), respectively, contrasted with their relatively low cellulose content of 22% for CGF and 15% for CP (Table 1). CGF is notable for its considerable protein content (22%), and CP is distinguished by its elevated starch levels (15%) (Table 1).

Investigations into pCA release demonstrated that CC produced 4.9 ± 0.4 mmol L^–1^ after alkali treatment and a much lower yield of 0.04 ± 0.01 mmol L^–1^ following XynZ treatment. In contrast, SCB yielded 2.9 ± 0.1 mmol L^–1^ of pCA after alkali treatment and 0.62 ± 0.02 mmol L^–1^ after XynZ treatment, whereas the previous literature shows SCB contains 1.3% FA and 1.8% pCA.^33^ In this sense, Miscanthus leaves also showed significant pCA release with 2.0 ± 0.2 mmol L^–1^ after alkali treatment or 0.44 ± 0.01 mmol L^–1^ using XynZ (Table 1).

Field-emission electron scanning microscopy (FESEM, Figure S1) provided insights into the morphologies of CP, CGF, and SCB following alkaline or enzymatic treatments. Alkaline treatments altered the morphology of CP, CGF, and SCB more than enzymatic treatments, which aligns with increased yields of FA and pCA after alkaline treatments. FESEM corroborates with the role of alkaline extraction in breaking down the starch granules on the CP surface, a process less pronounced with enzymatic treatment. In SCB, the alkaline treatment effectively exposed cellulose fibers due to the extraction of lignin and hemicellulose.^34^ Conversely, CGF is more diverse due to its origin as a wet milling byproduct, resulting in minimal morphological difference post-treatment, as observed under FESEM (Figure S1).

Following the results, wheat straw exhibited 0.81 mmol L^–1^ of FA and 0.49 mmol L^–1^ of pCA, while Miscanthus variants were documented with 1.4 mmol L^–1^ of FA and 2.0 mmol L^–1^ of pCA, corresponding to 0.4–0.8% FA and 1.0–1.8% pCA content.^12,35^ Other studies have reported HCAD content similar to our results, from barley (0.71 mmol L^–1^ of FA and 0.60 mmol L^–1^ of pCA), cocoa husk (0.03 mmol L^–1^ of FA), coffee husk (0.04 mmol L^–1^ of FA and 0.03 mmol L^–1^ of pCA), and rice bran (0.8 mmol L^–1^ of FA and 0.4 mmol L^–1^ of pCA) with concentrations ranging from 0.1 to 0.7 wt %.^6,36^

Interestingly, the alkali hydrolysates of red grape peel demonstrated the pCA release of 0.85 ± 0.06 mmol L^–1^ (Table 1), following Arnous and Meyer (2010), who reported high yields of pCA release from grape peels after the hydrolysis process.^37^ Although grapes differ markedly from other closely related dicots, that might indicate a distinctive evolution associated with resveratrol, biosynthesis from pCA might not be attached entirely to the plant cell wall of grape peels, as it is present in other lignocellulosic substrates herein reported.^37,38^

To the best of our knowledge, the prior literature has yet to report on the HCAD composition or extraction methodology for annatto bran, eucalyptus barks, and peanut hull (Table 1).

Finally, accordingly, to Table 1, the alkaline treatment was efficient to break ester bonds within the lignocellulosic matrix, leading to a higher HCAD release. In contrast, enzymatic methods are limited by the inherent plant biomass recalcitrance as well as the lack of dedicated enzyme cocktails and optimized ester-linked HCADs from lignocellulose. For the best of our knowledge, there are not specialized enzymatic solutions for efficiency HCAD extraction from lignocellulosic biomass. Alternately, the alkaline methods comprise an effective approach for achieving substantial HCAD yields in downstream applications, consistently outperforming enzymatic methods across all tested biomass types according to our study.

### Enzymatic Process Improvement

**Figure 1:**
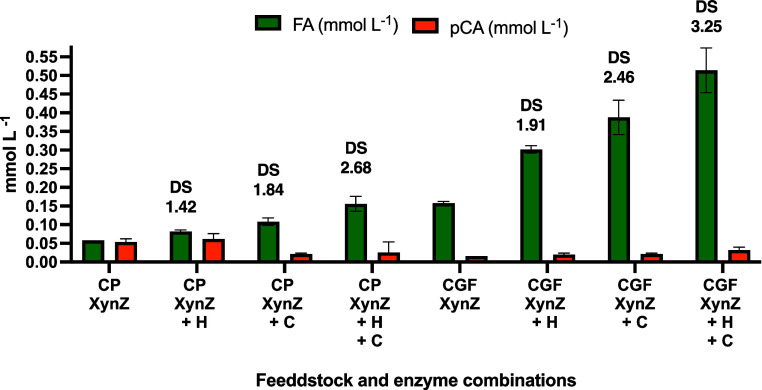
HCAD production after biomass hydrolysis using XynZ alone or in combination with Cellic Ctec2 (C) and/or Cellic Htec (H). All the essays were performed in triplicate. The degree of synergism (DS) represents the improvement of HCAD release with XynZ + Cocktails/XynZ only. No significant HCAD release was detected using commercial cocktails alone (data not shown).

Harnessing enzyme synergism is a promising approach to increase lignocellulose depolymerization and obtain higher HCAD yields, as shown in Table 1. Therefore, we explored the potential of enzymatic synergism via supplementation with Cellic CTec2 (cellulolytic) and Cellic Htec (hemicellulolytic) to increase HCAD release performed by XynZ (Figure 1).

The XynZ supplementation with both enzyme cocktails enhanced up to 3-fold FA release from CP and CGF, resulting in 0.16 ± 0.01 mmol L^–1^ and 0.51 ± 0.1 mmol^–1^ FA, respectively (Figure 1). Moreover, after evaluating the composition of these hydrolysates, it was possible to detect 1.9 ± 0.1 and 2.4 ± 0.1 g L^–1^ of total protein in CGF and CP hydrolysates and 27 ± 2 and 16 ± 2 g L^–1^ of total sugars post-enzymatic hydrolysis. These results demonstrate the potential of these hydrolysates as sources of protein and carbohydrates for further biorefinery applications. For instance, it is widely reported that developing enzymatic cocktails can substantially improve depolymerization and promote lignocellulose modification, but the focus on HCAD release improvement has been poorly evaluated in the literature.^16,48,49^

According to our data, alkaline methods remain an effective approach for achieving substantial HCAD yields in downstream applications, consistently outperforming enzymatic methods across all tested biomass types. For the best of our knowledge, there are no specialized enzymatic solutions to improve the efficiency of HCAD extraction directly from integral lignocellulosic biomass.

### Chemometric Analysis by Principal Component Analysis and Pearson Correlation

**Figure 2:**
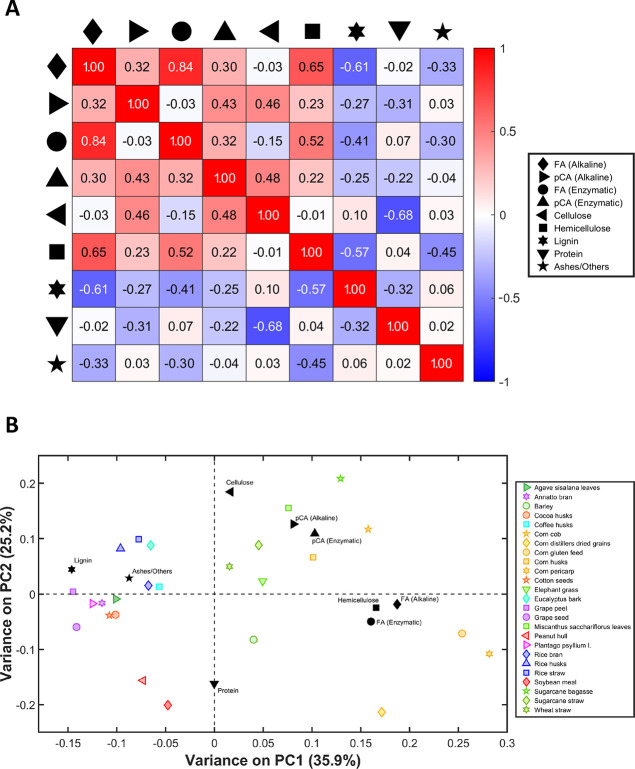
(A) Pearson correlation coefficient matrix of lignocellulosic composition of biomasses used in this study. This analysis measures a linear pairwise correlation between biomass components and the HCDA release. (B) PCA biplot of PC1 versus PC2, which comprises the sample, biomass composition, and HCAD release in a two-dimensional space. Scores icons present information related to biomass samples represented by colorful markers. Yellow (corn), light green (grasses), dark blue (rice), red, orange, purple, and pink (dicots) were considered the main groupings in the PCA that also have phylogenetic relationships. Loadings present information related to lignocellulosic biomass components and the HCAD release information axis, represented by black markers.

Principal Component Analysis is a multivariate technique to simplify data by reducing its dimensionality while preserving most of its variability. Therefore, Principal Component Analysis and Pearson correlation were applied as chemometric methods to explore the correlations, similarities, and differences among the different variables in biomasses related to their composition and HCAD release results (Figure 2A,B). Noteworthily, the principal components (PC) are organized in order of cumulative variance; i.e., PC1 presents more data information than PC2, and so on. Pearson correlation matrix is presented in Figure 2A, highlighting the pairwise parallels between variables such as hemicellulose, cellulose, pCA, and FA, supporting Principal Component Analysis findings and interpretation.

The first two principal components of the Principal Component Analysis model described 61.1% of the total data variation (Figure S2). It indicates that two dimensions characterize most of the information related to chemical composition and FA and pCA release (Table 1). PC1 versus PC2 biplots (Figure 2A) display scores representing different biomasses (colorful markers) and loadings (black markers) comprising the composition of these biomasses. Regarding treatment differences, Principal Component Analysis (Figure 2A) shows a high degree of similarity between FA and pCA obtained by both treatments. In other words, the enzymatic treatment using XynZ showed a pattern similar to the alkaline approach, as indicated by the strong positive correlation of 0.8 for FA and a moderate correlation of 0.4 for pCA (Figure 2B), despite variations in biomass among the samples tested.

Regarding the data variance explained by PC1, the negative axis (left side of Figure 2A) was primarily linked to the lignin and ash content. In contrast, the positive axis (right side of Figure 2A) is related to the other variables (cellulose, hemicellulose, pCA, and FA contents). Interestingly, correlation analysis confirms that the release of FA or pCA and the lignin content present a moderate negative correlation (between −0.6 and −0.3), indicating a potential reduction in FA and pCA concentration with increasing lignin content.

HCADs are aromatic compounds commonly associated with lignin since of the similarities in their chemical composition. The similarities might suggest that biomasses with a higher lignin content would result in higher FA and pCA release. However, the most accessible FA and pCA are likely the ones that act as cross-linkers with AX and can be extracted during alkaline and enzymatic treatments. Interestingly, according to our analyses, biomasses with lower lignin contents are likely more promising for FA and pCA production.

The data variance on PC1 also highlights that the hemicellulose content positively correlated with FA release in both treatments. This correlation was also shown by a Pearson correlation of 0.7 for alkaline treatment and 0.5 for enzymatic treatment. This positive correlation indicates that higher hemicellulose content is associated with higher FA release (Figure 2). In this sense, biomasses with high hemicellulose content tend to have reduced lignin content, such as CP, CGF, and DDGS.^13^ This correlation aligns with their respective functions and locations in the plants, especially concerning the plant’s stage of development and tissue and their lignin content.^13^ Likewise, in grasses, HCADs are primarily esterified to AX hemicellulose rather than being solely integrated into the lignin matrix. Therefore, biomass with a higher arabinoxylan content, such as CP and CGF, tends to release more HCADs. At the same time, reduced lignin content also facilitates the extraction of these components since lignin is the main barrier to biomass deconstruction.

Regarding the data variance described by PC2, the positive axis (upper side of Figure 2A) was predominantly influenced by the cellulose content and pCA released by both treatments. The data corroborated the Pearson correlation of 0.5 with both pCA alkaline and enzymatic treatments. In contrast, FA did not show the same trend, being close to zero in PC2 (horizontal dashed line in Figure 2A) and corroborated by the low values in the Pearson correlation (Figure 2B). Those findings suggest cellulose-rich biomass might yield higher pCA concentrations than biomasses with low cellulose content in this data set (Figure 2A,B). For example, this outcome was observed between CC (31.0% cellulose) and CP (15.1%) (Table 1).

Further exploring the data presented in Figure 2A shows that biomass originating from corn (yellow markers) exhibits the highest concentrations of FA for both treatments and they clustered together on the right side of the PCA biplot (Figure 2A). This clusterization indicates the correlation between high FA release and hemicellulose content. Accordingly, CC and corn husk, considered the most recalcitrant and cellulose-rich biomasses from corn, also possessed reasonable amounts of pCA (Table 1). Therefore, they clustered in the upper part of the graph closer to cellulose and pCA. Additionally, eucalyptus (dicot) and rice straw (monocot) are PC2 positive, justifying the elevated concentrations of pCA and high cellulose content (43.1 and 28.6%) after this treatment. The relationship between rice straw and husks with pCA is clearer in PC2 than PC3, as presented in Figure S2.

**Figure 3:**
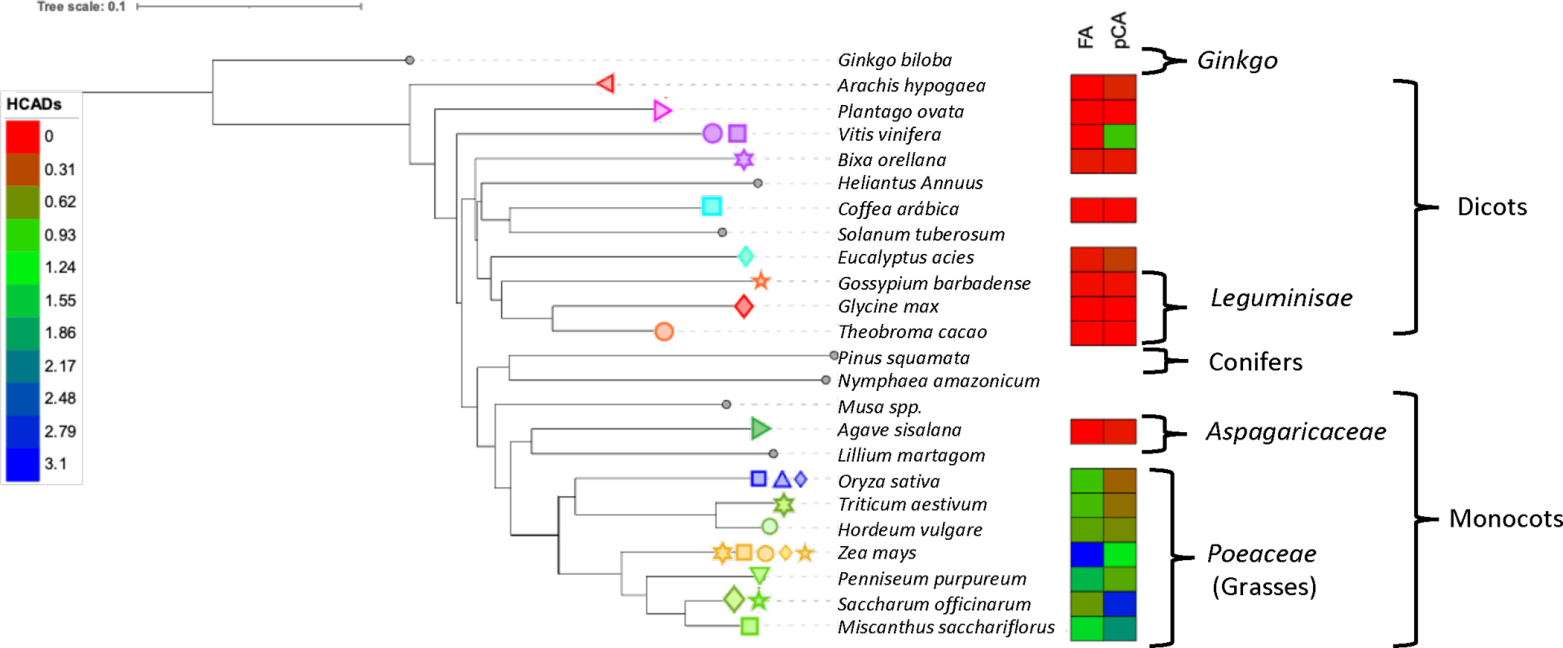
ITS phylogenetic tree among the industrial crops used in this work. The evolutionary history was inferred using the Neighbor-Joining using the Maximum Composite Likelihood method. The heatmap displays the amount of FA and pCA obtained in the present study in mmol L^–1^. The image was generated by inserting the phylogenetic tree into the Itol Tree of Life database, and the respective values are found in Table 1. In our study, the entries with gray icons were not evaluated based on their HCAD content. The average values of FA and pCA were considered when the plant species had more than one biomass sample in the data set of the present study.

Biomass with relatively high concentrations of AX displayed a positive correlation of HCAD release compared to biomass richer in other hemicellulose types such as galactomannans (Figure S3). According to the PCA biplot, the HCAD group and grasses display high concentrations of AX (green markers) (Figure S3). However, biomasses containing hemicelluloses like nondecorated xylans and galactomannans have not positively influenced HCAD release, grouping on the negative part of PCA 1 and 2 (lower left side of Figure S3). It is worth mentioning that hemicelluloses like xylans and homogalacturonans are preeminently found with their side chains methylated, which could hamper HCAD availability and content.^13^ These homogalacturonans are primarily abundant in dicots, whereas most AX-rich biomasses are monocots (Figure 3).

In addition to chemometric analysis, a phylogenetic tree was constructed to trace the evolutionary relationship of the biomasses, their HCAD content, and their botanic origins (Figure 3). Monocots, particularly those in the *Poales* order, like corn and sugar cane, exhibit higher HCAD release, in contrast with agave from the *Asparacaceae* order, which demonstrated negligible amounts of HCADs (Figure 3). Moreover, as reported in the literature, *Lilium bulbs*, a monocot from the *Liliales* order, exhibited a substantial free FA content of 0.3% (w/w), emphasizing the significant variation in HCAD accumulation and physiological role among plant taxa out of the *Poales* order.^50^

Overall, dicots display lower HCAD yields than grape peels, an exception in our study’s entire set of evaluated dicot biomasses. As explained earlier, the high resveratrol content in grape peels can corroborate our findings. Data from species found in the literature, such as *Ginkgo biloba*, sunflower, potato peels, and *Pinus* barks, provide a better evolutionary fitting for this analysis, with FA contents ranging from trace amounts to 0.1 wt %, highlighting the diversity of HCAD content in plant phylogeny.^51−53^

The similarity between Principal Component Analysis using compositional parameters and the phylogenetic relationships inferred from ITS sequences indicates that HCADs may play a significant role as phenotypic traits for crops’ ability to withstand environmental stresses and adapt evolutionarily. Overall, our research suggests that AX is the primary hemicellulose component contributing to the FA content in plant biomass, a conclusion drawn from comparing different biomass sources.

Our statistical analysis, employing Pearson’s Correlations and Principal Component Analysis, has revealed a noticeable correlation between pCA and cellulose content, a discovery not previously documented in the literature as reflected in the HCAD profiles of SCB, Miscanthus, and elephant grasses (Table 1). This correlation, although significant, should be interpreted with caution, recognizing that the correlation does not necessarily imply causation. The effects of cellulose and lignin on the composition and breakdown of HCADs require further investigation, particularly considering the uncertain impact of *p*-coumaroylation within these structural elements.^54^ It is known that pCA is implicated in the cross-linking within grass cell walls between their components, specifically through dimerization and ester-linking to lignin in the GAX matrix.^55^ Also, the variation of pCA in the lignin architecture of grasses lignocellulose serves as a terminator for lignin polymerization, conferring a more linear configuration to syringyl-type lignin, which is prevalent in grasses.^11,56,57^

### HCAD Extract Antioxidant Capacity

We scaled up the alkaline extraction from CGF, CC, and CP, resulting in hydrolysates with a range of 2.3–2.6 g L^–1^ FA and varying concentrations of pCA, from 1.8 g L^–1^ for CC to 0.1 g L^–1^ in CP (Table 2). After the extracts were dried via rotary evaporation, 1 kg of biomass yielded the following amount of HCADs: 7.3 g for CGF, 4.7 g for CC, and 10.5 g for CP (Table S2).

**Table 2:** Antioxidant Capacity of Each HCAD Extract

**biomass type**	**ORAC assay (μmol TE. g**^**–1**^**)**
**CGF**	2203 ± 8
**CC**	3012 ± 10
**CP**	3289 ± 14
**FA**	4417 ± 3
**pCA**	4413 ± 2
FA + pCA	4815 ± 1
**caffeic acid**	2.8 ± 0.1^b^
**chlorogenic acid**^a^	2.6 ± 0.1^b^
**isoquercetin**^a^	5.1 ± 0.1^b^
**quercetin**	5.5 ± 0.1^b^
	

a Positive experimental control.

b Data of pure compounds are expressed as relative Trolox (TE).

The antioxidant capacities of HCADs derived from lignocellulosic biomass, as reported in this study, underline the efficacy of alkaline hydrolysis in producing high-value bioactive compounds (Table 2). The antioxidant capacities of the CGF, CC, and CP extracts were notably high, ranging from 2203 ± 8 to 3289 ± 14 μmol of TE g^–1^ (Table 2). These values are substantially higher than those reported in recent studies where enzymatic and alkaline corn and sugar cane hydrolysates displayed ORAC values of 1050 and 1960 μmol TE g^–1^, respectively.^58,59^

The cost-effectiveness of using agro-industrial residues, paired with high antioxidant activity, presents a compelling case for the commercial application of these hydrolysates. A promising application is in the cosmetic sector, where natural antioxidants are increasingly favored over petrochemically based alternatives.^60^ Including FA derived from lignocellulosic biomasses and further demonstration of pCA as an alternative are interesting for more sustainable alternatives to impart antioxidant properties to cosmetic products. Moreover, integrating these extracts could augment the stability and efficacy of other active ingredients, such as ascorbic acid, vitamin E, and phloretin, thus enriching the product’s antioxidant matrix.^60^

### Whole-Cell Biotransformation of CGF Hydrolysate into Flavor Compound 4-VG

**Figure 4:**
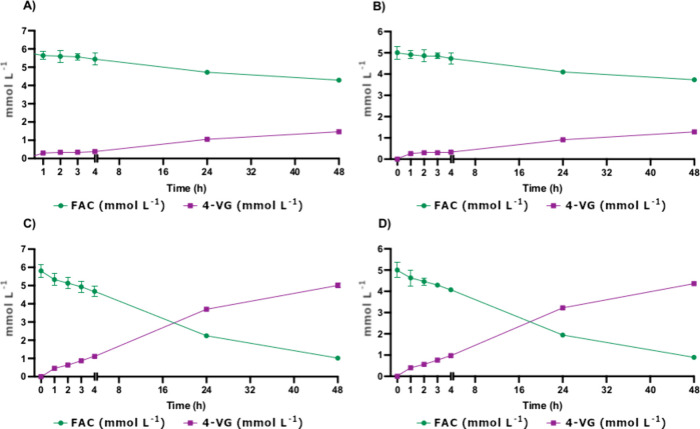
Amount of 4-VG obtained after the whole-cell biotransformation of CGF alkaline hydrolysate and 5 mM control FA during 48 h of fermentation. (A) WT Ethanol Red *S. cerevisiae* strain fermented on CGF hydrolysate. (B) WT Ethanol Red *S. cerevisiae* strain fermented on purified FA. (C) PDC mutant Ethanol Red *S. cerevisiae* strain fermented on the CGF hydrolysate. (D) PDC mutant Ethanol Red *S. cerevisiae* strain fermented on purified FA. The results are in mmol L ^–1^, and all of the essays were performed in triplicate.

Among the evaluated biomasses, the CGF hydrolysate was the most promising FA source in this study without further purification. Therefore, it was chosen as the material for transforming FA into 4-VG by using whole cells in one-pot reactions. A medium with 80% (v/v) CGF alkaline hydrolysate was benchmarked with synthetic medium with 5 mmol L^–1^ analytical grade FA where they were prepared and inoculated with either a control (*S. cerevisiae* Ethanol Red) or a 4-VG producing strain called *S. cerevisiae* Ethanol Red_PDC_B5, which overexpress a phenolic decarboxylase from *R. fluviale* LM-2. Figure 4 shows the levels of FA and 4-VG in the fermentation broth during the time, and the hydrolysate’s efficiency of conversion and fermentability was assessed for both strains.

The yeast mutant PDC_B5 outperformed the conversion of FA (from the hydrolysates) into 4-VG, significantly exceeding the results of the WT (Figure 4). Following a 48 h fermentation process in these hydrolysates, the PDC_B5 strain yielded 5.0 ± 0.2 mmol L^–1^ of 4-VG, substantially higher than the 1.5 ± 0.2 mmol L^–1^ obtained from the WT strain. This substantial 5.1-fold increase in the conversion yield by PDC_B5 is attributed to the integration of the PDC gene.^17^ Comparatively, the Basidiomycota *Rhodotorula mucilaginosa* yielded a maximum 4-VG concentration of 1.6 ± 0.1 g L^–1^ over 55 h from commercial grade FA.^61^

Therefore, the significant increase in 4-VG production and productivity in *Saccharomycetes* class yeast underlines the potential of integrating the PDC gene from other yeast species to improve specific enzyme activities relevant to phenolic compound bioconversion in industrial *S. cerevisiae* strains.^62^ Such genetically modified organisms could be scaled for industrial applications using agro-industrial hydrolysates, focusing on efficiency and rapid production of 4-VG, critical commercial parameters.

### Waste to Wealth: Corn Residue Hydrolysates and Their Potential Feasibility for Market Applications

In Brazil, the price of corn-related residues ranges between 59 and 118 USD per ton.^63^ Accordingly, a potential scenario exists where FA and pCA extracts from corn residues described in this study demonstrate the “waste to wealth” concept. For instance, 1 ton of CP could yield 10.5 kg of FA-containing dry extract, potentially valued at 9950 USD, compared to the current price of chemical companies supplying this compound in the USA in bulk quantities.^64,65^

Accordingly, our previously reported study where commercial FA was converted to the monolignol coniferyl alcohol using *Escherichia coli* as a biocatalyst of choice presented a minimum product selling price (MPSP) of 2100 USD per kg.^15^ Thus, given that the current price of 4-VG in bulk quantities in the United States is approximately 1911 USD per kg, the use of an inexpensive source of FA such as CGF alkaline hydrolysates and the yeast Ethanol Red-PDC_B5a shows promise for even greater commercial potential. However, further refinement and scaling of this technology could reduce reliance on the petrochemical resources of 4-VG and support global sustainability initiatives.

In fact, the relationships between the chemical compositions of lignocellulosic biomass and the extraction of high-value aromatic compounds are a field with many gaps yet to be elucidated. Notably, there is a direct association between hemicellulose content and FA release in grasses, and grasses rich in cellulose exhibit a significant increase in pCA release. Surprisingly, the lignin content negatively correlated with the release of HCADs after mild alkaline treatment, where all these results shed light on biomass complexity and these main components’ correlation. After these 28 biomasses were screened, corn-derived hydrolysates demonstrated high antioxidant capacity and excellent potential for whole-cell fermentation to produce 4-VG, among other chemicals, paving the way for the future development of a profitable biomass valorization process. Therefore, further research involved in technical and economic analyses to enhance our understanding of this complex interplay is highly endorsed.

## Supporting Information Available

Supplementary file 1: FESEM images of untreated, alkali-treated, and enzymatically treated biomasses; biomass sources and origins not previously reported; PCA: principal component analysis plots and biplots demonstrating variance and hemicellulose type differences; biotechnological applications: summary of HCAD content, extraction efficiencies, and antioxidant capacity of each extract; ITS sequences for phylogenetic analysis of studied biomass sources; complementary references (PDF)

## ABBREVIATIONS

AX: Arabinoxylan
CC: Corn Cob
CDW: Cell Dry Weight
CGF: Corn Gluten Feed
CP: Corn Pericarp
DDGS: Distiller’s Dried Grains with Solubles
FA: Ferulic Acid
GAX: Glucuronoarabinoxylan
HCAD: Hydroxycinnamic Acid Derivatives
ITS: Internal Transcribed Spacer
ORAC: Oxygen Radical Absorbance Capacity
PCA: Principal Component Analysis
pCA: *p*-Coumaric Acid
PDC: Phenolic Acid Decarboxylase
SCB: Sugar Cane Bagasse
S/G: Syringyl/Guaiacyl
TE: Trolox Equivalent
WT: Wild-Type
4-VG: 4-Vinylguaiacol
