# Supplementary material for: Hydroxycinnamic Acid Extraction from Multiple Lignocellulosic Sources: Correlations with Substrate Composition and Taxonomy for Flavoring and Antioxidant Applications
Source: J Agric Food Chem. 2024 Dec 5;72(50):28048–59. doi: 10.1021/acs.jafc.4c08540 (PMC11660217; doi:10.1021/acs.jafc.4c08540)
Supplement: Supplementary file 1 — jf4c08540_si_001.pdf [file jf4c08540_si_001.pdf]

Supplementary file:

Hydroxycinnamic Acid Extraction from Multiple Lignocellulosic Sources: Correlations with Substrate Composition and Taxonomy for Flavoring and Antioxidant Applications

*Robson Tramontina [a; b], Eupidio Scopel [c], Victor Gustavo Kelis Cardoso [c], Manoela Martins [d], Marcos Fellipe da Silva [d], Bárbara Flaibam [d], Marcos J. Salvador [a], Rosana Goldbeck [d], André Damasio [a], Fabio Marcio Squina [b] **

[a] Universidade Estadual de Campinas (UNICAMP), Departamento de Biologia Funcional e Molecular (BFM), 13083-859,Campinas, São Paulo, Brazil.

[b] Universidade de Sorocaba (UNISO), Laboratório de Ciências Moleculares, 18023-000, Sorocaba, São Paulo, Brazil.

[c] Universidade Estadual de Campinas (UNICAMP), Instituto de Química, 13083-970, Campinas, São Paulo, Brazil.

[d] Universidade Estadual de Campinas (UNICAMP), Escola de Engenharia de Alimentos, 13083-862, Campinas, São Paulo, Brazil.

* Email: fabio.squina@prof.uniso.br

1 -Morphological characterization after mild alkaline and XynZ treatment

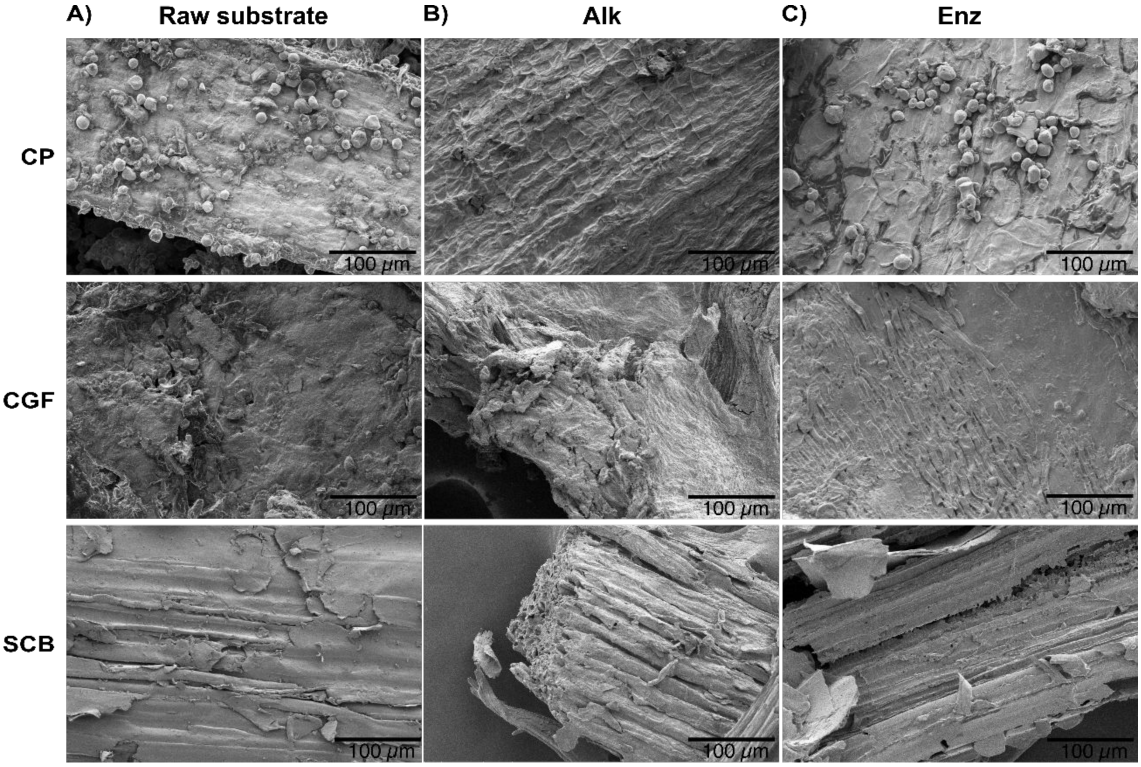

Figure S1. Field emission scanning electron microscopy (FESEM) was used to analyze the morphology of untreated, alkali-treated, and enzymatically biomasses. CP, CGF, and SCB: A) Raw substrates; B) after alkaline (Alk) treatment; and C) after enzymatic (Enz) treatment. Evaluating the chemical and morphological characteristics is crucial to better understanding the effectiveness of enzymatic and alkali treatments on different biomasses.

Table s1 – Biomass sources and origins not previously reported.

**Biomass**	**Source**
Agave sisalana leaves	[1] – Embrapa's germplasm bank, Paraiba – Brazil
Annatto bran	PASCHOINI AGRO LTDA – EPP – São Paulo – São Paulo – Brazil
Barley	L. CORDEIRO ALVES – São Paulo – São Paulo – Brazil
Cocoa husks	[2] – Bahia – Brazil (14°47″10.3'S, 39°16″40.6'W)
Coffee husks	[3] – Local farm, Boa Esperança, Minas Gerais – Brazil
Corn Cobs	INJEPLASTINDISTRIAL LTDA – São Paulo – São Paulo – Brazil
Corn gluten feed	Cargil, Campinas – São Paulo
Corn husk	Feira Nova Flour Industry, Pernambuco – Brazil
Cottonseed	L. CORDEIRO ALVES – São Paulo – São Paulo – Brazil
DDGS	[4] – Local market, Campinas – São Paulo – Brazil
Elephant grass	[5] – Institute of Animal Science, Nova Odessa – São Paulo – Brazil
Eucalyptus bark	[4] – Local market, Campinas –São Paulo – Brazil
Grape peel	ECONATURA PROD ECO E NATURAIS LTDA – Rio grande do Sul – Brazil
Grape seed	Viva Regenera – São Paulo –São Paulo – Brazil
Green Value lignin	Timothy D. H. Bugg – University of Warwick, Coventry – United Kingdom
Miscanthus sacchariflorus leaves	[5] – Institute of Animal Science, Nova Odessa – São Paulo – Brazil
Peanut hull	[4] – Local market, Campinas – São Paulo – Brazil
Plantago psyllium	INGREDIENTE STORE COMERCIO ONLINE LTDA – São Paulo – São Paulo – Brazil
Rice bran	JOSE GABRIEL GAMA MARMITT – São Paulo – São Paulo – Brazil
Rice husk	[6] – Local rice mil, Porto Ferreira – São Paulo – Brazil
Soybean meal	[4] – Local market, Campinas –São Paulo – Brazil
Sugarcane bagasse	[7] – Usina Vale do Rosário – São Paulo – Brazil
Sugarcane straw	[8] – Usina Ferrari – São Paulo – Brazil
Wheat arabinoxylan	[9] – Megazyme, Bray, Ireland
Wheat straw	[10] – Neil Dixon, MIB, Manchester – United Kingdom.
Wheat straw organosolv lignin	[11] - Timothy D. H. Bugg – University of Warwick, Coventry – United Kingdom

2 - PCA Analysis

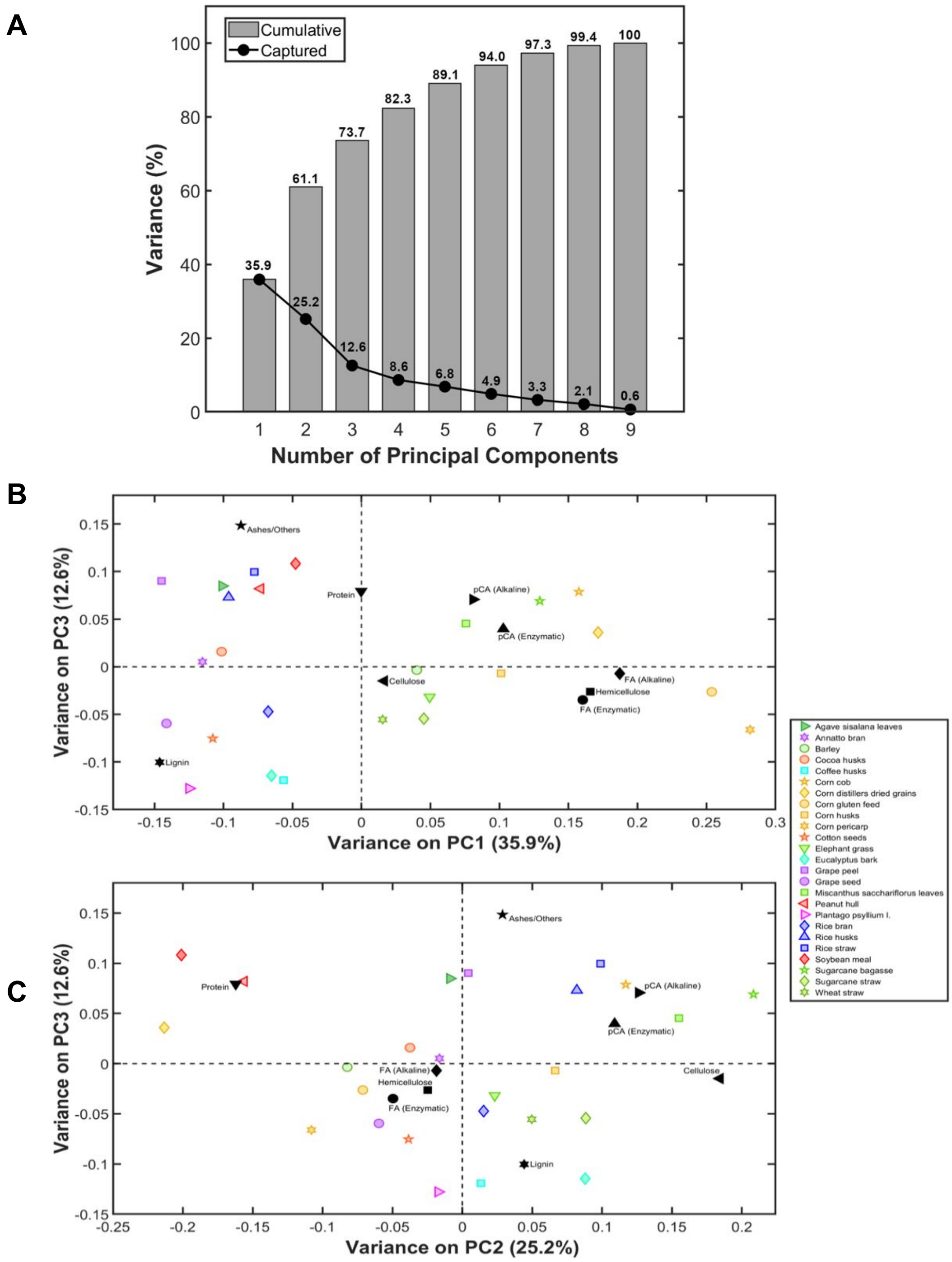

Figure S2. **(A)** The plot of captured and cumulative variance on each principal component. The first principal components describe the significant variance, whereas the last presents low information, usually related to residual information. **(B)** Biplot of PC1 versus PC3. **(C)** Biplot of PC2 versus PC3.

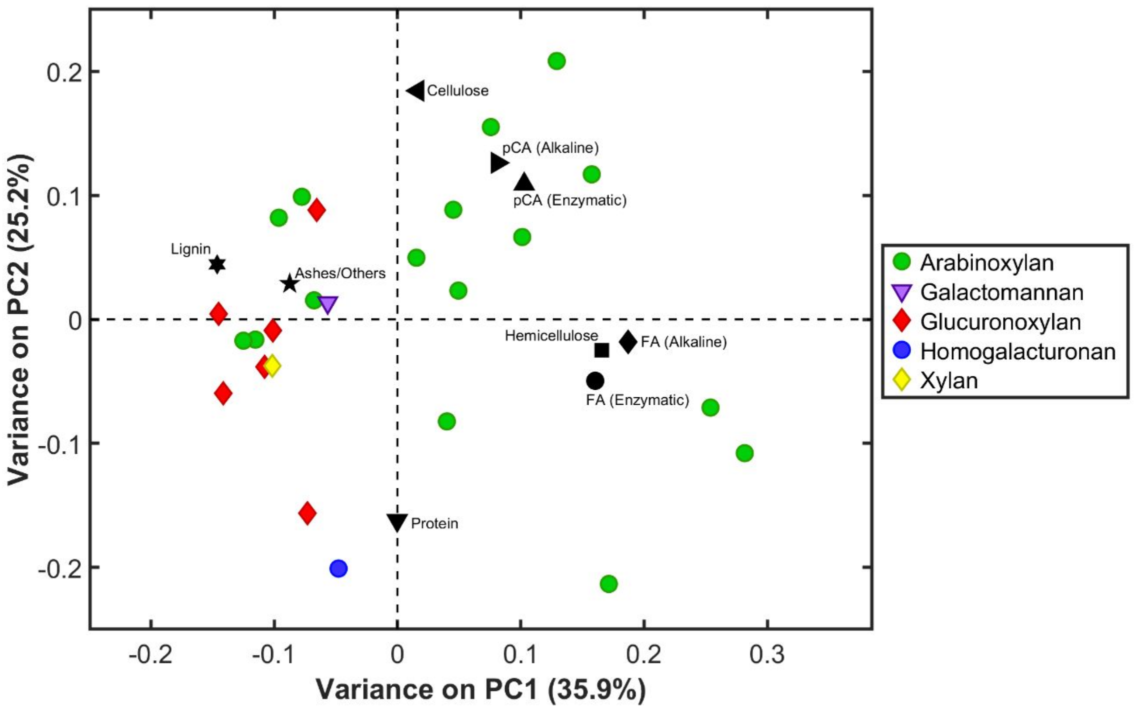

Figure S3. PCA biplot of PC1 versus PC2. This is the same PCA plot presented in Figure 2A, but each distinct color and shape in the scores plot represents different hemicellulose types, demonstrating the differences or similarities in their intrinsic characteristics.

3 – Biotechnological applications

Table S2: Summary of HCAD content and extraction efficiencies and the antioxidant capacity of each extract.

**Biomass Type**	**FA (g/Kg)**	**pCA (g/Kg)**	**Total HCAD (g/Kg)**	**Extract dry weight (g/Kg)**	**Extraction Efficiency (%)**	**ORAC assay (µmol TE g^{-1})**
CGF	23 ± 2.0	4.4 ± 0.2	27 ± 2.0	7 ± 3	27.1	2202 ± 8.2
CC	23 ± 4.0	18.2 ± 0.8	42 ± 4.0	5 ± 3	11.3	3012 ± 10.4
CP	25.8 ± 0.7	1.0 ± 0.3	27 ± 1.0	10 ± 2	39.2	3289 ± 13.5
FA	-	-	-	-	-	4417 ± 2.7
pCA	-	-	-	-	-	4413± 21.6
FA + pCA	-	-	-	-	-	4815.0 ± 0.6
Caffeic acid [a]	-	-	-	-	-	2.8 ± 0.1 [b]
Chlorogenic acid [a]	-	-	-	-	-	2.6 ± 0.1 [b]
Isoquercetin[a]	-	-	-	-	-	5.1 ± 0.1 [b]
Quercetin[a]	-	-	-	-	-	5.5 ± 0.1 [b]

a positive experimental control: b data of pure compounds are expressed as relative Trolox.

4 – ITS sequences

>*Agave_sisalana*
TCATTGTTGAGGCCCGAACGGATGATTGTGAACCTGTTAACGCATCCGATGGGGGCGGGGGT
GGGCGACATTTGCCGCACGCCGCCTGCCTCCTCGGGGCACCATGGCTGCCCCCGCCTTGCCTT
GCCGCGGGGTGGGGGCTGCGAACAACACCGGGCGCGGTGGGCGCCAAGGAACAGTGCTTCT
GGAGAGCAGCGTGCGTCGGACCGCCGAGGTGGGAAAGGCGCACGATGCGATCCTGCAATAT
CGTTAACTTTACGACTCTCGGCAACGGATATCTTGGCTCTCGCATCGATGAAGAACGTAGCGA
AATGCGATACTTGGTGTGAATTGCAGAATCCCGTGAACCATCGAGTCTTTGAACGCAAGTTGC
GCCCGAGGCTATCCGGCCGAGGGCACGCCTGCCTGGGCGTCACGCCTCGCGTCGCTCCGCGC
ACCCTGCCCCCACACAGAGCGGGCAGATGTGGGTGTGGATGTGGATATTGGCCCCCCGTGCC
TTGCGGTGGGTGGGTCGAAGTGCGGGCCGCCGGCCGGGTTGGACGCGGCGAGTGGTGGAC
GGACACGCTCGACGCTGAACGTCGCGAACCAAGCCCCGGCCTATGTGGCTCATGCAGGAAC
CCAATCCGAGTGGCGCTCACGAGCGCCCTCGGACAACGACCCCAGGTCAGGCGGGACCACCC
GCTGAGTTTAAGCATATCAATAA

>*Bixa_orellana*
GAAGGATCATTGTCGAAACCCTGCATAGCAGGACAACTCGTGAACCGTAGAATGCAACAAGG
GGGGCGAGAAGAGCGCCCCCCAACCGGTCGATCAAGCACTTGCGCCTTGCGCGCTCGTGCTT
TGAGTTGGCCTAACAACAAAACCCGGCGCGAGCCGCGCCAAGGTCGTCGGAACCAAAAAGA
GCAGGCACCCGTTGCCGCTGATAGCGCGCGCGATGGGTTGCTGGCACTCTTTCATGTGAGAA
ATGACTCTCGGCAACGGATATCTCGGCTCTCGCATCGATGAAGAGCGTAGCGAAATGCGATAC
TTGGTGTGAATTGCAGAATCCCGTGAACCATCGAGTCTTTGAACGCAAGTTGCGCCCCAAGCC
GTCAGGCCGAGGGCACGTCTGCCTGGGTGTCACGCATCACGTCCCACCAAAAATCCCTCCACT
CCTTGGAGGAGTGAGGCGCTTTGTGTTGTGGGCGGATATTGGCCTCCCGTGCGCACCCGCTC
GCGGTTGGCCAAAACGCGAGTCCCCGTTGAATCGGTATGAAGGCTAGCGGTGGTCCGTTGCG
CCCTCGAAACTTGCCTTGAGTGCCATTCCTCGGTATGGATCTCCCCGACCCTGATGCAGTCGTT
TGCATAGGCAGTTGCGACCCC

>*H_vulgare*
TCGTGACCCTGACCAAAACAGACCGTGCTCGCGTCATCCAATCCTCCGACGATGGCATTGTTC
GTCGTTCGGCCAATTCCTCGACCGCCTCCACTCCTAGGAGCGGGGGCTCGTGGTAAAAGAACC
CACGGCGCCGAAGGCGTCAAGGAACACTGTGCCTAACCCGGGGAGATGGCTAGCTTGCTGGT
CGTCACCTGTGTTGCAAATATATTTAATCCACACGACTCTCGGCAACGGATATCTCGGCTCTCG
CATCGATGAAGAACGTAGCGAAATGCGATACCTGGTGTGAATTGCAGAATCCCGCGAACCAT
CGAGTCTTTGAACGCAAGTTGCGCCCGAGGCCACTCGGCCGAGGGCACGCCTGCCTGGGCGT
CACGCCAAAACACGCTCCCAACCACCCTCTTCGGGAATTGGGATGCGGCATATGGTCCCTCGT
CCTGCAAGGGGCGGTGGGCCGAAGATCGGGCTGCCGGCGTACCGCGTCGGACACAGCGCAT
GGTGGGCGTCCTTGCTTTATCAATGCAGTGCATCCGACGCGTAGACGGCATCATGGCCTCGAA
ACGACCCATCGAACGAAGTGCACGTCGCTTCGACC

>*Theobroma_cacao*
GCATCGATGAAGAACGTAGCGAAATGCGATACTTGGTGTGAATTGCAGAATCCCGTGAACCA
TCGAGTCTTTGAACGCAAGTTGCGCCCAAAGCCATTAGGCTGAGGGCACGCCTGCCTGGGTG
TCACACATCGTCACCCCTTCCTCCACACTTAACCAGTTAAATGTGAGGCAGTGGGTGAAAGTTG
ACCTCCCGCGAGCCAGTTCCTCGTGGTTGGTTGAAAAGCAAGTTCGGGGCGGAGTCCCCCGC
GATAACGGTGGATGAGCCCACGCTCGAGACCAATCGTGCCTGTGGGACTCCGACGTAACGGA

CTTATCGACCCCACACGCGCCCTCTGTGCAAACCGAGTGTGCCATCTACGAGACCTCAGGTCA
GGCNGGGCTACCCGCTGAGTTTAAGCATATCAATAAGCGGAGGA

>*Coffea_arabica*
CTGCGGAAGGATCATTGTCGAATCCTGCATAGCAGATGACCGCGAACTCGTGTAATAGTCGG
GCGTCGGGGCGGGGGCGGTGAGGCCGAAACCTCTCCTCCCTCCCCGTCGCTCCCCGCGCGCT
CGTCGTGCGGACCAACAACCCAACCCCGGCGCGGAAAGCGCCAAGGAAAACTCAAAAGATCG
CTCGGCCCCCGACCGCCCCGTCCGCGGAGCGCGGGAGGGGATGCCGCGGCGTCTGTCGTAAC
CAAAACGACTCTCGGCAACGGATATCTCGGCTCTCGCATCGATGAAGAACGTAGCGAAATGC
GATACTTGGTGTGAATTGCAGAATCCCGCGAACCATCGAGTCTTTGAACGCAAGTTGCGCCCG
AAGCCTTTAGGCCGAGGGCACGTCTGCCTGGGCGTCACGCATCGCGTCGCCACCCCCCTCCCG
CGGGGGCGGCGGAGACTGGCCTCCCGTGCCCCCGGGCGCGGCCGGCCTAAACGCGAGTCCT
CGGCGGGGGACGTCACGACCAGTGGTGGTTGAGTCCCTCAACTCGAGTCCTTGTCGTGCCGTT
AGACCACCCGCCGCATTCGGGGCTCCGACGACCCTGAAGAGAGTTGCTCTCATCTCGACGGC
GACCCCAGGTCAGGCGGGATTACCCGCTGAGTTTAAGCATATCA

>*Zea_mays*
CCCCCCCGAACCTCCCGCGGGGAAGGGGGGGCCGCGAAAAAGAACCCACGGCGCCCCGGGC
GCCAAGGAACACCAGTACTACCTCCTGCCCCGCGGAGCGGTCGGCCCGCCTTCCGCTCCCAGG
GCAGCGGTTACACCTTAATCGACACGACTCTCGGCAACGGATATCTCGGCTCTCGCATCGATG
AAGAACGTAGCAAAATGCGATACCTGGTGTGAATTGCAGAATCCCGCGAACCATCGAGTTTTT
GAACGCAAGTTGCGCCCGAAGCCTTCTGGCGGAGGGCACGTCTGCCTGGGCGTCACGCCAAA
AGACACTCCCAACACCCCCCCGCGGGGCGAGGGACGTGGCGTCTGGCCCCCCGCGCCGCAGG
GCGAGGTGGGKCGAAGCAGGGGCTGCCGGCGAACCGCGCCGGGCGCAGCACGTGGTGGGC
GACATCAAGTTGTTGTTCTCGGTGCAGCGTCCCGGCGCGCGGCCGGCCATRCGGCCCTAAGG
ACCCATCGAGCGACCGAGCTTGCCCTCGGACCGCG

>*Gossypium_barbadense*
TCGAAACCTGCCTAGCAGAACGACCCGCGAACGCGTTGCAAACAACACCGGAGGTGGTGCGG
GTGCATCCTCGCCTCTCGCCACCCCCGTGTCTCGGAGCGGTCAGTCTCGTCGTCCCTTTGCCCG
TCGGGTGGGGTGAGATGCCGGGATCAACCTCTTCGAGGCAAAGCGAACAAACCCCCGGCGC
GAATCGCGCCAAGGAATCGAAACGAAAGAAGGGGCATGTCTTCTGTCGCCGCACCGTTCGCG
GTGTCGATGCTTCAGTGATGTTGCTCTCTTGTCGCAAAATATACAGAACGACTCTCGGCAACG
GATATCTCGGCTCTCGCATCGATGAAGAACGTAGCGAAATGCGATACTTGGTGTGAATTGCAG
AATCCCGTGAACCATCGAGTCTTTGAACGCAAGTTGCGCCCCAAGCCATTAGGCCGAGGGCAC
GTCTGCCTGGGTGTCACGCATCGTCGCCCCCATCCAACCATGAGCCCTCGAGCCTCGGTTGGA
CCGCGGGCGGAAATTGGCCTCCCGTGCGCTCACAGCCAGCGGTTGGCCTAAATTCGAGTCCTC
GACGACATCATCGTCGCGACGATCGGTGGTAATGCTGCAAGCAACCTCGTTCGGAGTCGTGC
GCGTCCGTCGATCGAGACCCTTGAACCCTTTCGGCATCGCAAGGACGGTGCTCGCA

>*Pennisetum_purpureum*
TCGTGACCCTTAAACAAAACAGACCGTGAACATGTCACCCATGCCGCTCGGGCTTCTGCTCGG
GCTAGGCCTCGACCTTCTTTTAGAGGGAAGGGGTCGCAAAAGAACCCACGGCGCCGAAGGCG
TCAAGGAACACTTATATTGCCTTGCCCGGGGTTGTGGTCGGCCTGCCGAACGCACCTCGTGCA
GCGATGCTATCTTAATCCACACGACTCTCGGCAACGGATATCTCGGCTCTCGCATCGATGAAG
AACGTAGCAAAATGCGATACCTGGTGTGAATTGCAGAATCCCGCGAACCATCGAGTTTTTGAA
CGCAAGTTGCGCCCGAGGCCTTCTGGCTGAGGGCACGTCTGCCTGGGCGTCACGCCAAAAGA

CACTCCCAACCCATCCGTGGGGAAGGATGTGGTGTTTGGCCCCCCGTGCCGTAAGGTGGGGT
GGGCCGAAGTTGGGGCCGCCGGCGTAACATGCCGAGCACCGCACGTGGTGGGCGACATACA
GTTGTTCTCGGTGCAGTGTCTCGGCTAGTAGTCGGCGTGTTGGCCTAAATGACCCATGACGAC
CGTAGCGCTTTGTCGCTCGGACC

>*Eucalyptus_acies*
CATTGTCGAATCCTGCCAAGCAGAATGACCAGAGAACCGGTAACAAACTCAACGGGGGCGGC
GGGCTCAGCCCGACGTCCCCCTCGACGCCGAGGATTTGGCTCGGGCGCCCCAGGGCGCTCGG
CCCCGTCCYCGGCGGCGCAACGAACCCCGGCGCGGAATGCGCCAAGGAACTYGAACAAGAGT
GCGATGCTCCCGCCGCCCCATACACGGTGCGCGCGCGGGAYGCCATGCAATCTCATATTAGTC
ATAACGACTCTCGGCAACGGATATCTCGGCTCTCGCATCGATGAAGAACGTAGCGAACTGCGA
TACTTGGTGTGAATTGCAGAATCCCGTGAACCATCGAGTCTTTGAACGCAAGTTGCGCCCGAA
GCCTTCGGGTCGAGGGCACGTTTGCCTGGGTGTCACACATGGCGTTGCCCCTAATCCCCCTCG
CCCTCTCAACCGGGCGAGCGGGGACYTGGGCGCGTAAGATGGCCTCCCGCGACGACCACGTC
CCGGCTGGCCCAAAATCGAGCGTCGGAGCGATCAGCACCACGACATTCGGTGGTTGATTAGA
CCCCAATGATCAATGTCGCGCGTGCCGCTCATGCGCACGCTCTGCGAATCTGCTCCTTACCAAC
GCGACCCCA

>*Vitis_vinifera*
CGTAACAAGGTTTCCGTAGGTGAACCTGCGGAAGGATCATTGTCGAACCTCCGAATGACCCGC
GAACACGTTACAACTCTCAGGGGGGGCGGAGCGTGGGCGCGAGCCCGCCGCTTTCGTCGCCC
CTCCCCCCGTTCAGGAGGTCCCGGGAGCCTCGCCCCTCATNGTGCCCAGCGCACGGGCGAGG
GGCGACCCATGGCGGCCCTCTTGAGCGAACAACAAACCCCTGCGCGGAACGCGCCAAGGAAC
CTAACAGCGAAGCAACGCGCTCCCGTTGCCCCGGTCCCGATCAGGCGCGTCGGGGAGTCGTC
GTCGTTTCATCAAACACAAACGACTCTCAGCAAATGATATCTAGGCTCTTGCATTGATGAAGAA
TGTAGCAAAATGCGATACTTGGTGTGAATTGCAGAATCCCGTGAATCATTGAGTCTTTGAACG
CAAGTTACGCTNGAAGCCATTAGGCCGAGGGCACGCCTGCTTGGGCGTCATGCACCCGTCGC
CCCCCCACCTCCCTCCCCCCCGAGGCCCTGCACTCGGGGACGAGAGGCAGAGAGGGGGCGGA
CATTGGCCTCCCGTGGGCGCCCCAGTCCGTTGTTAGCCAAAAATCGGTCCCCCGGCAACGTAC
GCCACAACGAGCGGTGGATTCGCACGACTTGGCGTCACGTNGCCTCAGGGCCCCCCCTCGAG
ACCCTGGATAGCAACCCCAGGTCAGGCAGGACCACCCGCTGAGTTTAAGCATATCAATAA

>*Miscanthus_sacchariflorus*
TTTCCGTAGGTGAACCTGCGGAAGGATCATTGTCGTGACCCTTAAACAAAACAGACCGCGAAC
GAGTCCCTCGTGCCGCCGAGCTTCGGCTCGGCATTAGGTCCCCGAGCTCCGTCCCGGGGCGG
AGGGGCCACAACAGAACCCACGGCGCCTTAGGCGTCAAGGAACACTTATATTGCCTTGCTCG
GCGGAGCGGTCGGCCTGCCTTCCGCTCCCCGCGCAGCGATGATATCTTAATCCACATGACTCT
CGGCAACGGATATCTCGGCTCTCGCATCGATGAAGAACGTAGCAAAATGCGATACCTGGTGT
GAATTGCAGAATCCCGCGAACCATCGAGTTTTTGAACGCAAGTTGCGCCCGAGGCCTTCTGGC
CGAGGGCACGTCTGCCTGGGCGTCACGCCAAAAGACACTCCCAACCCACCCAAGAGGGGGAG
GGACGTGGTGTTTGGCCCCCCGCGCCGCAGGGCGTGGTGGGCCGAAGTTGGGGCTGCCGGC
GAATCGTGTCGGGCACAGCACGTGGTGGGCGACACTCAGTTGTTCTCGGTGCAGTGCCCCGG
CAGTCGGCCGGCGCATCGGCCCTAAGGACCCATGTAGCACCGCAGCGCATCGCCGCTCGGAC
CGCGACCCCAGGTCAGTCGGGACTACCCGCTGAGTTTAA

>*Arachis_hypogaea*

GAATTCCTAGTAAGCGCGAGTCATCAGCTCGCGTTGACTACGTCCCTGCCCTTTGTACACACCG
CCCGTCGCTCCTACCGATTGAATGGTCCGGTGAAGTGTTCGGATCGCGGCGACGTGGGCGGT
TCGCTGCCGGCGACGTTGTGAGAAGTCCACTGAACCTTATCATTTAGAGGAAGGAGAAGTCG
TAACAAGGTTTCCGTAGGTGAACCTGCGGAAGGATCATTGTCGATGCCGCACAAACCAGGATT
GACGCGCGAACGAGTCCACAAACACCCGAGGCGGGGAAGGGCCGGCCGTGCGCGGCCGGC
GCCCCGTCTCAAACAAGAACAAAACCCCGGCGCGGAAAGCGCCAAGGAAGCCAAACGTTTCT
GCTCTCCCCGCCGGCTTCCGGAGACGGCATCCGGTCGGGCGAGGAGTGACCACAAGAGTTAA
AGAACGACTCTCGGCAACGGATATCTCGCTCTTGCATCGATGAAGAACGTAGCGAAATGCGAT
ACTTGGTGTGAATTGCAAGAATCCCGTGAACCATCGAGTCTTTGAACGCAAGTTGCGCCCGAA
GCCCTTAGGCTGGAGGGCACGCCTGCCTGGGTGTCAACCAAAAGGCGCCCCCCCGTCTCGCC
CGTCCCAGGGCACGGGGGAGGGGGCGAACGTTGGCCTCCCGGGAGCCCCTGGCTCGCGGTT
GGTTCAAAGAGACGGGCTCTTGGTGGGGAGCGGCACCGCGGCAGATGGTGGTCGAGAACAA
CCCTCGTGGCCAGTCGCGCGCGCCTCTCCCCCGGTTCAAGGCACGGCGACCCGCGGGCGACG
TGGATCGTCCCGAGCGCGACCTCAGGTCAGGCGGGGCTACCCGCTGAGTTTAAGCATATCAA
TAAGCGGAGGAAAAGAAACTAACGAGGATTCCCCTAGTAACGGCGAGCGAACCGGGAAGAG
CCCAGCATGAGAATCGGTCGCCCCTGGCGTCTGAATTGTAGTCTGGAGAAGCGTCCTCAGTG
GCGGACCGGGCCGAAGTCCCCTGGAAGGTGGCGCCAGAGAGGGTGAGAGCCCCGTTGTGCC
CGGACCCTGTCGCACCACGAGGCGCTGTTTGCGAGTCGGTTGTTTGGGAATGCAAGCCCTAAT
CGGGCGGTAAATTCCGTTCCAAGGCTAAATACTGGCGTGAGACCGATAGCGAAAAGTACCGC
GAGGGAAAGAAGAAAAGGACTTTGAAAAGAGAGTCAAAGAGTGCTTGAAATTGTCGGGAGG
GAAGCGGATGGGGGCCGGCGATGCGCCCCGGTCGGATGTGGAACGGCGACGCTGGTCCGCC
AATCGACTCGGGGCGTCGACCGACGCGGATTGCAACGGTGGCCCAAGCCCGGGCCGTCGATA
GGCCCGCTGGATACGTCATCGTTGCGATTGTGGAAGGCAGCGCGCGCCCGCTGGCGTGCTTC
GGCACCTGCGCGCTCCGGGCGTCGGCCTGTGGGCTCCCCATTCGGCCCGTCTTGAAACACGG
ACCAAGGAGTCTGACATGTGTGCGAGTCAACGGGTGAATAAACCCGCGGGGCGCAAGTAAG
CTAATTGGCGGGATCC

>*Plantago_ovata*
ACGTGGGCGGTTCGCTGCCCGCGACGTCGCGAGAAGTCCACTGAACCTTATCATTTAGAGGA
AGGAGAAGTCGTAACAAGGTTTCCGTAGGTGAACCTGCGGAAGGATCATTGTCGATATCTGA
AAAGTAGACCTGTGAACACGTGTTTAACATGAACGGTGCCTTGTTGGGCCAGAGACATCTTGC
TTGACGAGGCACCGTGCCTGCTTGGTGCTAGCACCTTGTGGGCTAACGAAACCCGGCGCGGT
AAGCGTCAAGGAAAACAAATTAGAAGCGTTGCCCTTGCAGCTCCCGTTCGCGGTGTGGCTGT
GGGGATGCAGCGTATCTTGAAAGTCAAAACGACTCTCGGCAACGGATATCTTGGTTCTCGCAT
CGATGAAGAACGTAGCGAAATGCGATACTTGGTGTGAATTGCAGAATCCCGTGAACCATCGA
GTCTTTGAACGCAAGTTGCGCCCGACGCCTTCGGGCTGAGGGCACGCCTGCCTGGGCGTCAC
GCATCGCGTCGCCCCCTCCCAAACCCATGTGGTTCGGTGATGGGGCGGACAATGGCTTCCCGT
TAGCTCGGTTAGCCTAAAAAGGATCCCTCAACGATGGATGTCACAACCAGTGGTGGTTGAAA
GATCATTGGTGCTGTTGTGCTTCACCCTGTCGCTTGCTAGGGCATCATCATAAACCAACGGCGT
GAAATGCGCCTTCGACCGCGACCCCAGGTCAGACGGGACTACCCGCTGAGTTTAAGCATATCA
ATAAGCGGTGGAGA

>*Oryza_sativa*
GCCGTGACCCTGACCAAAACAGACCGCGAACGCGTCACCCCTGCCCGCCGAGCGCTCGCGCG
CGAGGCAACCGAGGCCCCCGGGCCGCAACAGAACCCACGGCGCCGACGGCGTCAAGGAACA
CAGCGATACGCCCCGCGCCGGCCCGGTCGGCCCTGGCCGTCCGGCGGCGCGGCGCGATACCA
CGAGCTAAATCCACACGACTCTCGGCAACGGATATCTCGGCTCTCGCATCGATGAAGAACGTA

GCGAAATGCGATACCTGGTGTGAATTGCAGAATCCCGTGAACCATCGAGTCTTTGAACGCAAG
TTGCGCCCGAGGCCATCCGGCCGAGGGCACGCCTGCCTGGGCGTCACGCCAAAAGACGCTCC
ACGCGCCCCCCCTATCCGGGAGGGCGCGGGGACGCGGTGTCTGGCCCCCCGCGCCTCGCGGC
GCGGCGGGCCGAAGCTCGGGCTGCCGGCGAAGCGTGCCGGGCACAGCGCATGGTGGACAG
CTCGCGCTGGCTCTAGGCCGCAGTGCACCCCGGCGCGCGGCCGGCGCGATGGCCCCTCAGGA
CCCAAACGCACCGAGAGCGAACGCCTCGGACC

>*Glycine_max*
GTGAACTGCGGAAGGATCATTGTCGATGCCTCACAATCAGATTGACCCGCGAACTTGTTTATT
CATCTACCGTCGGGAGGGAGGGGATGACCACGGCGCCCCGTGCGCCCGGCCTCCTCGTCCTC
GCGACAAACACAAACCCCGGCGCTTCGTGCGCCAAGGAACTCAAATCTGTTAAGTGCGACTCC
CGGGGGCCCGGAGACGGTGTCCCGCGGGAGTCGTCACGACACAACATTTACATACAATGACT
CTCGGCAACGGATATCTCGGCTCTTGCATCGATGAAGAACGTAGCGAAATGCGATACTTGGTG
TGAATTGCAGAATCCCGTGAACCATCGAGTCTTTGAACGCAAGTTGCGCCCGAAGCCATTAGG
CCGAGGGCACGCCTGCCTGGGTGTCACACATCGTTTCCCCAACGCAAACATGTAACAATGTTG
CTGCGCGGGGTGTATGCTGACCTCCCGCGAGCACCCGCCTCGTGGTTGGTTGAAATCTGGGTT
CATGGCCGACTTCGCCGTGATAAAATGGTGGATGAGCCACGCTCGAGACCAATCACGTGCGA
GCCGGTCAGTTCTGGACCCATCGACGACCCTTTGCGTGCACGCACGCTCCCAACGAGACCTCA
GGTCAGGCGGGGCTACCCGCTGAG

>*Saccharum_officinarum*
GTCCCCCGAGGTCCGTCCTGAGCGGAGGGGCCACAACAGAACCCACGGCGCCTTAGGCGTCA
AGGAGCACCTATATTGCCCTGCGCGGTGGAGCGGTCGGCCCGCCTTCCGCTCCCCGGGCAGC
GATGATATCTTAATCGACACGACTCTCGGCAACGGATATCTCGGCTCTCGCATCGATGAAGAA
CGTAGCAAAATGCGATACCTGGTGTGAATTGCAGAATCCCGCGAACCATCGAGTTTTTGAACG
CAAGTTGCGCCCGAGGCCTTCTGGCCGAGGGCACGTCTGCCTGGGCGTCACGCCAAAAGACA
CTCCCAACCCACCCGAGGGGGAGGGACGTGGTGTTTGGTCCCCCGTGCCGCAGGGCGCGGTG
GGCCGAAGTTGGGGCTGCCGGCGAATCGTGTCGGGCACAGCACGTGGTGGGCGACACTCAG
TTGTTCTCGGTGCAGCGCCCCGGCACGCGGCCGGCGCATCGGCCCTAAGGACCCATGGAGCA
ACGCAGCGCATAGCCGCTCTTTTCCGCGCGACCCGGTCAGTCGGGACTACGCCGCGAGTAGG
G

>*Triticum_aestivum*
TCGTGACCCTGACCAAAACAGACCGCGCACGCGTCATCCAATCCGTCGGTGACGGCACCGTCC
GTCGCTCGGCCTATGCCTCGACCACCTCCCCTCCATCGGAGAGGGTGGGGGCTCGAGGCAAA
AGAACCCACGGCGCCGAAGGCGTCAAGGAACACTGTGCCTAACACGGGGCGCATGGCTAGCT
TGCTAGCTGTGACTCGTGTTGCAAAGCTATTTAATCCACACGACTCTCGGCAACGGATATCTCG
GCTCTCGCATCGATGAAGAACGTAGCGAAATGCGATACCTGGTGTGAATTGCAGAATCCCGC
GAACCATCGAGTCTTTGAACGCAAGTTGCGCCCGAGGCCACTCGGCCGAGGGCACGCCTGCC
TGGGCGTCACGCCAAAACAGGTTCCNACANCCCTCATTGGGAATCGGGATGCGGCATCTGGT
CCCTCGTCTCGCAAGGGACGGTGGACCGAAGTTAGGGCTGCCGGCGTACCGTGTCGAACACA
GCGCATGGTGGGCGTCTTTGCTTTATCAACTGCAGTGCATACGACGCGTAGCCGGCATTATGG
CCTCANAACGACCCAACAAACGTAGCGCACGTCGCTTCGACC

>*Lilium_martagon*
TCGAGAATCGATTGAGACACCGCGAACCTGTAAACGGATGATACCGTGTCGGGCGGGCGTTA
TGCCCGCCCAACTCGGGACCTCGCATCGTGTCCGTGGCCGCCTCGGAGCGTTTCGGGCACGAT

TTGCGGGGGACGAACGAAACCCCGGCACGGCCTGTGCCAAGGAACATATGTCAGGACGGAC
GTGTGTCAATGCCTCAGTGGTGGGGCGACGTCCGCTCTCTATTTATACGACTCTCGGCAACGG
ATATCTCGGCTCTCGCATCGATGAAGAACGTAGCGAAATGCGATACTTGGTGTGAATTGCAGA
ATCCCGTGAACCATCGAGTCTTTGAACGCAAGTTGCGCCCGAGGCCTTTCGGTTGAGGGCACG
CCTGCCTGGGCGTCACGCCTTGTTTCGCTCTGTGCCCATGCTCTTTCGGGGGCGGTCATGGAT
GCGGAGATTGGCCCTCCGTGCCTCGTGTGCGGCGGGCTTAAGTGCGGGCTGTCGGTGTCGGG
ATGGGCACGACGAGTGGTGGACGGAGCACCAGCAGGATGTTGTGGTCCCCTGTCGCCTTAAG
GGGCTCAAGAGACCCGGACTAGGCGAGCCGCCCTCCGTACGAGGAGGGCGAGCCGTCTCGC
AGG

>*Nymphaea_amazonum*
TTGTTCCCTATTGGATAGACTTGCGAACATGTTATCCCTCATGCGGAGGGGAGCATCCGCGCC
TTCACACGGTGTGGTGCTTTCCTTTGCTACTGGCTTTTGCCTTTGCTCTTGCCATTTGTCTCCATT
GGGGATGGCGGTGTCTTGAGCAAGTGCAAGGCCAAACTTAACAAAAAATCGGCGTTTTTAAG
CGCCAAGGAACTTTTTGATTGAAAGGAGAGGGCATCCCCCCACACGAAAGTGTGGGTGGAGA
TATGCCATTTCCCCTTCCATTATAACGACTCTCGGCAACGATATCTTGGCTCCCGCCACGATGA
AGAACGTAGCGAAATGCGATACTTGGTGTGAATTGCAGAATCCCGTGAATCATCGAGTTTTTG
AACGCAAGTTGCGTCCGAGGCCATTTGGCTAAGGGCACGCCTGCCTGGGCGTCAAGCTTCGC
ATCGCCCTTCCCATGTCCTTCTGCTTGCGAGCAAAGGCTGGGGTAGAGCGGAGGACTGGCCTT
CGGCGTCGGCCTTTGAGGCGCGCCGTTGGCTGAAACATTCCGGGCTTACGACGATCAGTTGAT
GGGCGCAACAAGCGGTGGATTTCCCAGTGAGTTGTGCCTCAGCTGATCGAGAAGGCCTACGG
GACTCTTGAGGCAAGCGGCTTAGGTTTCTTGCTTCGGCCTTC

>*Pinus_squamata*
CCCCCCTTTTTGCAAAGGCATGGGGTTCGGGGGGGCTTTTTTGAGCCCCTTCCCTTTAGCGAGT
GGGTTAGGGAGTGCGGTTGTTTCTCTTGTTGCGACGCATTGTACGTGGGGTGGCTTTTAGCCA
TTTCTCGTGGGGGTGCGATTTTTTTGGTAACACACGGAAACGACTCTCGGCAACGGATATCTC
AGCTCTTGTTACGATGAAGAACGTAGCGAAATGCGATACTTAGTGTGAATTGCAGAATCCTGT
GAATCATCGAGTTTTTGAACGCAATTTGCGCCCGAGGCTTCGGTCGAGGGCACGTCTGTCTGG
GCGTCGCATTCCAATCAAACGCGCCCCCTGCAA
TATGCTAGGGAGCAGCGGACGTGGTCGTCCGTGCCCAACGCGGTGCGGTTGGCTGAAATGTG
GTAGGCGATGTTTCGTGGCATGCGTCGGCAAGCGGTGATCTTGTCCCCTTGGTTGGGCAGTCG
GCGTTAGCCGATGCGGGCTCTGTGTGGCATCCCTGGAACTTGCCTTGCTCTCTCTTGTCCTCCC
ATTGGGTAGGGCGGATTTAGCTCCAACTTGCG

>*Solanum_tuberosum*
GGATCATTGTCGAACCCTGCAAAACAGAACGACCCGCGAACACGTTTTAAACACTTGGGGGC
GCTTGCGCCCTCCCGTCCCCGACGACTTGCGCGCTTGCGCGCTCGTTTTTGGGGCCAACCAAC
GAACCCCGGCGCGGAAAGCGCCAAGGAATACTAAAATCGGCAGCCCTCCCCTCGCGCCCCGT
TCGCGGATCGCCCGGGGGGACGCGCGCTGCTCTTTAAACACAAACGACTCTCGGCAACGGAT
ATCTCGGCTCTCGCATCGATGAAGAACGTAGCGAAATGCGATACTTGGTGTGAATTGCAGAAT
CCCGTGAACCATCGAGTCTTTGAACGCAAGTTGCGCCCGAAGCCATTAGGCCGAGGGCACGT
CTGCCTGGGCGTCACGCATCGCGTCACCCCCCGCACATTGCGMGGGGGCGGAAGCTGGCCTC
CCGTGCGCCCCGAGCGCACGGCTGGCCTAAATGCGAGTCCACATCAACCGACGTCGCGGCAA
GTGGTGGTTGAAGCTCAACTCTCTCTCGTTGTCGCGGCTACCGCCCGTCGCGCGTCCGGACTC
CCAGACCCTGATTGCGCCATGGCGCTCCGACCGCGACCCCAGGTCACCGACCAGCAACGCACC
TCCGGGGGGGTGCCAAAA

>*Helianthus_annuus*
TCGAACCCTGCACAGCAGAACGACCCGTGAACAAGTTAACACATCTGGCCTTGCCGGGACCG
AAGCATTTGTTTCGGCCCTTGTGAGTCCTTGTCGACGTGCGTTCATGCATGGACCATACCTTTG
GTTTTGTCATGGATGTCATGTTGACAAAATAACAAACCCCCGGCACGAGATGTGCCAAGGAAA
ACCAAAATTAAAGAACACGTGCTGTTGCGCCCCGTTCGCGGTGTGCGCGCTGTTCGTGGCTTC
TTTGTAAACTTAAAACGACTCTCGGCAACGGATATCTCGGCTCACGCATCGATGAAGAACGTA
GCAAAATGCGATACTTGGTGTGAATTGCAGAATCCCGTGAACCATCGAGTTTTTGAACGCAAG
TTGCGCCCGAAGCCATTCGGTTGAGGGCACGTCTGCCTGGGCGTCACGCATCACGTCGCCCCC
ACCAGGCATCCCCTATAGGGCTGTCTTGTGTTGGGGCGGAGATTGGTCTCCCGTGCCCATGGC
GTGGTTGGCCTAAATAGGAGTCTCCTCGCGAGGGACGCACGGCTAGTGGTGGTTGATAAGAC
AGTCGTCTCGTGTCGTGCGTTTACTTTCTTGAGAGTAGATGCTCTTAAAGTACCCTGATGTGTT
GTCTTATGACGATGCTTCGA

>*Musa banksii*
GAAGGAGAAGTCGTAACAAGGTTTCCGTAGGTGAACCTGCGGAAGGATCATTGTCGAGACCC
ACTGACGAGGACGACCGTGAATGCGTCAACGATTGCTCGTCGGGCTCGTCCCGACAACACCCC
GAATGTCGGTTTGCCCTCGGGCGGGACGATCGAGGGGATGAACTACCAACCCCGGCGCGGAT
AGCGCCAAGGAACACGAACATCGAAGTCGGAGGGCCTCGCTGCATGCAGGCTACGATTCCGA
CGGTGACCCCATTGGACGACTCTCGGCAACGGATATCTCGGCTCTCGCATCGATGAAGAACGT
AGCGAAATGCGATACCTGGTGTGAATTGCAGAATCCCGTGAACCATCGAGTCTTTGAACGCAA
GTTGCGCCCGAGGCCATCCGGCTAAGGGCACGCCTGCCTGGGCGTCACGCTTTCGACGCTTCG
TCGTTGCCCCCTCGGGGGGTGGGGGCGAACGCGGAGGATGGCCCCCCGTGCCGGAAGGTGT
GGTTGGCTGAAGAGCGGGCCGTTGGTGGTTGTCGAACACGACGCGTGGTGGATGCCTTGTGC
GAGCCGTACGTCGTGCCTTCGGGACCCGGGCGAGGCCTCGAGGACCCAAGTCGTGGTGCGA
GTCGATGCCACGGACCGCGACCCCAGGTCAGGTGGGGCTACCCGCTGA

> *Ginkgo_biloba*
CAAGTTGCGCCCGAAGCCTTGCGAGGAAANGGCATGTGTGTATGCGTCTCACGCATCACCTCC
CCCTGCACTTCCACGGTGGGGCTGCGAAGATGGTCGTCGGTGTCCCCTAGGGGCGGCTGACT
CATCATCATGTGGTGGTCATCTCTCCATGCCCTTGATCAATGCCTCAGCCTCTCGTTGCGTGATC
GATCTACACTACTCAGGAGTATCTGGCGAGCCTCTCCGCAAACAACTTCTAACTCTAACCTCGG
TCGCTATGCGCACACAGGGCAGTCATTCAGATGTTGTGCGCGAGCCTGGGTCACGCAATATCA
CCCTTTGAGTTTATGCATATCACTAAACATAGGAAAAGACACTTACAACCATTCCCCTAATAAT
GGCGAGCGAACCTTGAAAATCCCAGCATGAAAATCTAGTGATCGTGTTGCCCAAATTGTACTC
TGGAGAAGCGT

5 - Complementary references

1. Raya FT, Marone MP, Carvalho LM, Rabelo SC, de Paula MS, Campanari MFZ, et al. Extreme physiology: Biomass and transcriptional profiling of three abandoned Agave cultivars. Ind Crops Prod [Internet]. 2021;172:114043. Available from: https://linkinghub.elsevier.com/retrieve/pii/S0926669021008086
2. Moretti LK, Ramos KK, Ávila PF, Goldbeck R, Vieira JB, Efraim P. Influence of cocoa varieties on carbohydrate composition and enzymatic activity of cocoa pulp. Food Res Int [Internet]. 2023;173:113393. Available from: https://linkinghub.elsevier.com/retrieve/pii/S0963996923009389
3. Ávila PF, Goldbeck R. Fractionating process of lignocellulosic biomass for the enzymatic production of short-chain cello-oligosaccharides. Ind Crops Prod [Internet]. 2022;178:114671. Available from: https://linkinghub.elsevier.com/retrieve/pii/S0926669022001546
4. Flaibam B, Goldbeck R. Effects of enzymes on protein extraction and post-extraction hydrolysis of non-animal agro-industrial wastes to obtain inputs for cultured meat. Food Bioprod Process [Internet]. 2024;143:117–27. Available from: https://linkinghub.elsevier.com/retrieve/pii/S096030852300127X
5. Scopel E, Rezende CA. Biorefinery on-demand: Modulating pretreatments to recover lignin, hemicellulose, and extractives as co-products during ethanol production. Ind Crops Prod [Internet]. 2021;163:113336. Available from: https://linkinghub.elsevier.com/retrieve/pii/S092666902100100X
6. Moreira BR, Breitkreitz MC, Simister R, McQueen-Mason SJ, Gomez LD, Rezende CA. Improved hydrolysis yields and silica recovery by design of experiments applied to acid-alkali pretreatment in rice husks. Ind Crops Prod [Internet]. 2021;170:113676. Available from: https://linkinghub.elsevier.com/retrieve/pii/S0926669021004404
7. Tramontina R, Franco Cairo JPL, Liberato M V., Mandelli F, Sousa A, Santos S, et al. The Coptotermes gestroi aldo–keto reductase: a multipurpose enzyme for biorefinery applications. Biotechnol Biofuels [Internet]. 2017;10:4. Available from: http://biotechnologyforbiofuels.biomedcentral.com/articles/10.1186/s13068-016-0688-6
8. Brenelli LB, Figueiredo FL, Damasio A, Franco TT, Rabelo SC. An integrated approach to obtain xylo-oligosaccharides from sugarcane straw: From lab to pilot scale. Bioresour Technol [Internet]. 2020;313:123637. Available from: https://linkinghub.elsevier.com/retrieve/pii/S0960852420309093
9. Megazyme. Megazyme Arabinoxylan (Wheat Flour; Low Viscosity) [Internet]. 2024 [cited 2024 Apr 12]. Available from: https://www.megazyme.com/arabinoxylan-wheat-flour-low-viscosity
10. Tramontina R, Galman JL, Parmeggiani F, Derrington SR, Bugg TDH, Turner NJ, et al. Consolidated production of coniferol and other high-value aromatic alcohols directly from lignocellulosic biomass. Green Chem [Internet]. 2020;22:144–52. Available from: http://pubs.rsc.org/en/Content/ArticleLanding/2019/GC/C9GC02359C
11. Williamson JJ, Bahrin N, Hardiman EM, Bugg TDH. Production of Substituted Styrene Bioproducts from Lignin and Lignocellulose Using Engineered Pseudomonas putida KT2440. Biotechnol J. 2020;1900571:1–8.
